# Non-Suicidal Self-Injury in Autism Spectrum Disorder: A Systematic Review of Associated Factors and Management Difficulties

**DOI:** 10.3390/jcm15031254

**Published:** 2026-02-04

**Authors:** Julia Valentina Coll-Oltra, Ártemis Lambrou-Martínez, Julio A. Camacho-Ruiz, Rosa M. Limiñana-Gras, Carmen M. Galvez-Sánchez

**Affiliations:** 1 Department of Clinical Psychology, Reina Sofía General University Hospital, Multiprofessional Teaching Unit in Mental Health (UDMSM), 30003 Murcia, Spain; juliavalentina.collo@um.es; 2 Department of Psychiatry, Virgen de la Arrixaca University Clinical Hospital, Multiprofessional Teaching Unit in Mental Health (UDMSM), 30120 Murcia, Spain; artemis.lambroum@um.es; 3 Department of Personality, Evaluation and Psychological Treatment, Faculty of Psychology and Speech Therapy, University of Murcia, Building 31, 30100 Murcia, Spain; julioangel.camachor@um.es (J.A.C.-R.); liminana@um.es (R.M.L.-G.); 4 Foundation Project Man Jaén, 23002 Jaén, Spain; 5 Regional International Campus of Excellence (CEIR) Mare Nostrum Campus (CMN), 30100 Murcia, Spain; 6 Assisted Reproduction Unit, Quironsalud Murcia Medical Center, 30008 Murcia, Spain

**Keywords:** autism, non-suicidal self-injury, autism spectrum disorder, emotion regulation, associated factors, early intervention

## Abstract

**Background**: Non-suicidal self-injury (NSSI) is defined as the intentional damage to one’s body tissue without suicidal intent and for reasons that are not socially sanctioned. While NSSI has been widely studied in the general population, its clinical correlates and management in autism spectrum disorder (ASD) remain less clearly characterized, and it is often conflated with self-injurious behavior (SIB) described within restricted and repetitive behaviors (RRBs). In individuals with ASD, NSSI may be associated with emotional, behavioral, cognitive, social, medical, and demographic factors, and it differs from SIB typically observed among individuals with severe intellectual disabilities. **Methods**: A systematic review was conducted in accordance with PRISMA guidelines. Studies published between 2000 and 2025 that assessed NSSI in individuals with a formal ASD diagnosis were included. Etiological/clinical correlates, explanatory mechanisms, and management challenges were examined. Sixteen studies were selected from PubMed, Scopus, and Web of Science. Findings were synthesized using narrative and thematic approaches. **Results:** The prevalence of NSSI among individuals with ASD ranged from 24% to 50%. Associated factors included emotion dysregulation (including alexithymia and affective distress), behavioral dysregulation (e.g., impulsivity/hyperactivity and aggression), sensory processing difficulties, communication and social impairments, and medical comorbidities (i.e., gastrointestinal and sleep problems), with preliminary evidence also implicating perinatal factors. NSSI was linked to emotion regulation, sensation seeking, and social communication processes. Early intervention and parental involvement were identified as protective factors. **Conclusions**: NSSI in ASD is a complex, multifactorial phenomenon frequently linked to emotion-regulation needs. Affective imbalance represents a central—though not exclusive—pathway. The review supports standardized terminology, function-based assessment, and clearer differentiation from SIB/RRBs, with implications for individualized interventions and sustained monitoring in persistent or severe cases. Routine screening for medical and sensory contributors may further improve case management and reduce preventable clinical burden.

## 1. Introduction

Non-suicidal self-injury (NSSI) is defined as behaviors in which an individual intentionally harms the body without overt suicidal intent and for reasons that are not socially sanctioned, as defined by the International Society for the Study of Self-Injury [1,2,3].

The most common NSSI comprises behaviors such as cutting, burning, scratching, and self-battery [2]. Rates of NSSI are high and concerning among youth and young adults, across both clinical and non-clinical populations, with a lifetime prevalence that ranges from 12 to 46% in adolescent and young adult populations [2].

It is relevant to note that NSSI is included in the broad concept of self-injurious behavior (SIB), that widely refers to aggressive behaviors directed toward oneself. The most frequent SIB is head banging, often in combination with biting, scratching, or hair pulling [4], and it is commonly associated with intellectual disability and ASD. Nevertheless, it has been reported to be at least two to three times more common in children with autism than in children with broadly defined intellectual disabilities, with prevalence rates estimated at 35–50% among children with ASD compared to 5–17% among children with intellectual disabilities [5].

Although SIB is not exclusive to autism, it constitutes one of the most severe comorbid behavioral problems in children with ASD, and the wide range of prevalence mentioned before may be explained due to differences in the sampled populations, shifting diagnostic trends, or the lack of standardization in the operational definitions of SIB, so classifications of SIB/non-SIB may vary across studies, particularly concerning the frequency or severity of its expression [5].

For its part, ASD is defined as a neurodevelopmental condition of neurobiological origin characterized by difficulties in social reciprocity and nonverbal communication, along with a pattern of repetitive and restricted behaviors, interests, and activities [6]. ASD typically manifests in childhood, is a lifelong condition, and is commonly associated with additional neurodevelopmental or mental health issues. Although autistic symptoms have been traditionally viewed as lacking functional purpose, individuals with ASD have reported that stereotyped behaviors often serve a regulatory function, particularly in stress management [7,8,9].

ASD is described as a complex and highly heterogeneous condition. Its complexity stems from the interplay of its diverse etiology, varied clinical presentation, and the differing evolutionary trajectories observed across lifespan. Furthermore, its manifestations are significantly influenced by factors such as age, gender, and comorbidities. Dr. Stephen Shore summarizes the characteristic “heterogeneity” of ASD in one sentence: “If you’ve met one person with autism, you’ve met one person with autism” [10].

ASD prevalence is estimated in 1 of 68 children, affecting males 3 times more than females—although clinical essays suggest that it could be 4 times more prevalent in males [7,11]—and the reported sex ratio of ASD diagnosis typically varies from 4:1 to 2.0–2.6:1 [12]. Nevertheless, it has been reported that there is an underdiagnosis of ASD in females, leading to a late detection or a mistaken diagnosis. This disparity is influenced by several diagnostic biases, such as gender differences in clinical manifestations and cognitive abilities [12].

Diagnosing ASD requires a multidisciplinary and comprehensive evaluation. Firstly, the assessment process should integrate several sources of information, including parental reports, direct observation, interaction with the child, and the clinician’s expert judgment. To increase reliability, clinicians commonly use standardized diagnostic tools, such as Autism Diagnostic Interview-Revised (ADI-R) and Autism Diagnostic Observation Schedule-Second Edition (ADOS-2) [13,14]. However, beyond the primary diagnosis of ASD, it is crucial to detect comorbidities associated with ASD, as in approximately 90% of cases, ASD is associated with other neurodevelopmental or mental health issues [9].

Thus, in early childhood, ASD is commonly associated with high rates of comorbidity: more than half of cases are associated with one or more other neurodevelopmental alterations. Specific neurodevelopmental impairments are highly prevalent: 30–40% of children present symptoms of hyperactivity, inattention, and impulsivity. Furthermore, approximately one-third of individuals with ASD exhibit severe impairments in language development, and around one-third are also diagnosed with an intellectual disability [11], affecting significantly functional adaptation. The remaining two-thirds, with intellectual capacity within the normal range, present a high degree of dependence [6,7]. During adolescence and adulthood, especially in individuals with ASD and higher cognitive and communicative abilities, conditions such as eating disorders, psychotic symptoms, gender dysphoria, and behavioral alterations are notably heightened. In addition, in this group of individuals, there is an increased association with emotional disorders and suicidal ideation or suicidal behaviors, which may lead to completed suicide [11].

As research on self-injury in ASD has traditionally focused on SIB within the broader framework of RRBs, the potential for NSSI to occur in the context of ASD has often been overlooked [15]. This bias is partly reinforced by how RRBs have been operationalized in commonly used assessment tools, in which SIB is frequently embedded as a subtype of repetitive behavior (e.g., the SIB subscale within the Repetitive Behavior Scale–Revised, RBS-R) [16]. As a result, much of the ASD literature has prioritized highly repetitive and stereotyped forms of self-injury, while comparatively less attention has been paid to NSSI as a distinct construct that may involve different functional mechanisms and clinical correlates [17]. Importantly, this conceptual overlap risks obscuring NSSI presentations more consistent with those described in the broader clinical literature, such as self-injury used to modulate aversive internal states, relieve emotional tension, or communicate distress—functions that have been repeatedly reported by autistic individuals [18]. Indeed, prior work has highlighted the scarcity of ASD-focused research explicitly examining NSSI despite elevated rates of established vulnerability factors in this population (e.g., depression and difficulties in emotion regulation), suggesting that NSSI in autistic individuals has remained under-investigated [17]. Consequently, inconsistent terminology and the interchangeable use of “SIB”, “self-harm”, and “NSSI” may contribute to ongoing conceptual ambiguity, limiting the identification of NSSI-specific risk markers and constraining the development of tailored assessment and intervention strategies in ASD [18].

Given the overlap between the terms referring to self-harm along scientific literature, in the present systematic review, the term NSSI has been used to refer to any form of self-harm without suicidal intent, including what some studies refer to as SIB.

Based on previous findings, the primary objective of this systematic review is to synthesize the available evidence on NSSI in individuals with a formal diagnosis of ASD by (1) identifying and organizing reported etiological and clinical correlates across six domains—emotional, behavioral, cognitive, social, medical, and demographic—and (2) characterizing key clinical management challenges, including the chronicity/persistence, severity and topography, and functional characteristics of NSSI, as well as barriers to effective assessment and intervention delivery in this population.

Taken together, these objectives enable a more precise conceptualization of NSSI in ASD and highlight the unique contribution of the present review. In contrast to prior ASD-focused work that has primarily framed SIB within RRBs, we explicitly distinguish NSSI as a separate construct and highlight its potential role in emotion regulation. We further advance the field by structuring evidence across six domains—emotional, behavioral, cognitive, social, medical, and demographic—and by detailing clinical complexities such as chronicity, severity, topography, and functional characteristics. Together, these contributions address a persistent conceptual and empirical gap and provide a foundation for improved assessment and tailored interventions for autistic individuals who engage in NSSI.

## 2. Materials and Methods

### 2.1. Protocol and Search Strategy

This systematic review was conducted in accordance with the guidelines of the Preferred Reporting Items for Systematic Reviews and Meta-Analyses (PRISMA) statement [19]. The PICO question guiding this review was: Which factors are associated with the onset and maintenance of NSSI in individuals with ASD, and what are the main clinical consequences? The PRISMA checklist for scoping systematic reviews is provided in the Supplementary Material (Table S1). The review protocol has been pre-registered in PROSPERO [CRD420251276506] and the Open Science Framework (OSF) (https://osf.io/9f4bx/overview?view_only=f8e55c031c034874976788140484bf0c accessed on 30 September 2025).

Researchers J.V.C.-O. and Á.L.-M. independently performed the literature search across the PubMed, Scopus, and Web of Science (WOS) databases. The search strategy was constructed using a combination of medical subject headings (MeSH) terms and free-text terms, focusing on the population (ASD) and the phenomenon (NSSI), and incorporating specific filters to exclude suicidal behavior and studies published before 2000. The search query used across all databases was the following: (“autism spectrum disorder” [Mesh] OR autism* OR ASD OR “Asperger syndrome”) AND (“self-injurious behavior” [Mesh] OR “non-suicidal self injury” OR “non suicidal self-injury” OR “self injury” OR “self-injury” OR “self harm” OR “self-harm” OR “self mutilation”) NOT (“suicidal behavior”[Mesh] OR suicid*). The concept “[Mesh]” was only used on Pubmed, as other databases did not require it. The last search was performed on 25 December 2025.

The screening and selection processes were conducted independently by two researchers (J.V.C.-O. and Á.L-M.) and proceeded in two phases. In the initial screening, the two researchers independently screened the titles and abstracts of the retrieved articles to eliminate irrelevant research based on the inclusion and exclusion criteria. Secondly, full-text analysis of the pre-selected articles were retrieved and independently analyzed in depth by the two researchers to determine their final eligibility.

In both phases, any initial discrepancy regarding the inclusion or exclusion of an article was resolved by consensus between all researchers. The overall process was conducted under the supervision of C.M.G.-S. A detailed overview of the study selection procedure is provided in the PRISMA flow diagram.

### 2.2. Eligibility Criteria

The inclusion and exclusion criteria were based on the PICO framework. As inclusion criteria we considered (1) participants with a formal diagnosis of ASD (DSM-4, DSM-5, DSM-5-TR, ICD-10/11 or validated clinical diagnosis), (2) peer-reviewed studies assessing NSSI, self-injury, or self-harm explicitly documented as without suicidal intent or that offer a detailed operationalization of self-harming behavior that allowed us to exclude suicidal intent based on the context, (3) studies analyzing associated factors, explanatory mechanisms, or clinical implications, (4) empirical studies (quantitative, qualitative, or mixed-methods) published in peer-reviewed journals including a comparison group that is either ASD without SIB or a group without an ASD diagnosis, and (5) articles published in English or Spanish, from 2000 onwards. Likewise, articles were excluded if they were (1) samples based on ASD traits, (2) studies focused exclusively on suicidal behavior or self-aggressive behavior with suicidal intent, (3) isolated case reports lacking an analysis of associated factors, (4) review articles, meta-analyses, case studies, editorials, letters, theses, or protocols, or (5) duplicated studies.

### 2.3. Data Extraction and Quality Assessment

Two researchers (J.V.C.-O. and Á.L.-M.) independently extracted the characteristics, methodologies, and main findings of each included article. Disagreements were resolved through discussion and, when necessary, consultation with a third reviewer. Inter-rater reliability was assessed using Cohen’s κ. Agreement was substantial, with κ = 0.72 for title/abstract screening and κ = 0.75 for full-text screening. The overall process was conducted under the supervision of C.M.G.-S. The following data were retrieved for inclusion in the study characteristics table: first author, year of publication, country, and study design, sample size and demographics (i.e., age, sex, control sample), ASD diagnostic criteria, instrument used, included variables, clinical consequences, and main results. The study characteristics are shown in Table 1. Data on etiological and clinical correlates were systematically extracted and organized into six domains, with the full synthesis presented in Table 2.

In order to evaluate the quality of the selected articles, both, Á.L.-M. and J.V.C.-O. independently evaluated the risk of bias (ROB) in each study. Study quality and risk of bias was assessed using the Joanna Briggs Institute (JBI) Critical Appraisal Checklist for Analytical Cross-Sectional and Cohort Studies (8 and 11 items respectively) [20]. Each criterion was rated as Yes, No, Unclear, or Not applicable. For the overall appraisal, only “Yes” ratings were considered criteria met, whereas “No” and “Unclear” were treated as not met. In cross-sectional studies, those meeting 0–4 criteria were classified as high risk of bias/low quality, those meeting 5–7 criteria as moderate risk of bias/moderate quality, and those meeting 8 criteria as low risk of bias/high quality. In cohort studies, those meeting 0–6 criteria were classified as high risk of bias/low quality, those meeting 7–9 criteria as moderate risk of bias/moderate quality, and those meeting 10–11 criteria as low risk of bias/high quality. These thresholds were applied consistently across studies and are reported to facilitate interpretation of the quality appraisal results. To enhance methodological rigor, the risk-of-bias assessment was independently replicated by two reviewers (J.A.C.-R., and R.M.L.-G.), and consensus ratings were established through discussion (with arbitration by a third reviewer when required). As in previous occasions, the analysis of limitations was discussed by all authors, who made the final decision together. The overall process was conducted under the supervision of C.M.G.-S. Overall risk-of-bias classifications for each included study are reported in Table 3, whereas Table 4 and Table 5 provides the item-level appraisal results by JBI criterion/domain.

### 2.4. Data Synthesis

The final synthesis was performed narratively and structured thematically. The main objectives, methodology, and clinical relevance of the findings of each included study were checked. This analysis of study characteristics and quality—following the PRISMA guidelines—was conducted to better understand the link between ASD and NSSI and provide clinical implications to be considered when managing NSSI in individuals with ASD.

## 3. Results

The results of this systematic review are presented in a structured and thematic manner to provide a comprehensive understanding of the factors associated with NSSI in individuals with ASD. First, the characteristics of the included studies are summarized, highlighting their design, sample size, and geographical distribution. Subsequently, the associated factors are categorized into six domains: emotional, behavioral, cognitive, social, medical, and demographic. Each domain is analyzed in detail, emphasizing the key findings and their implications. Finally, the clinical implications and challenges related to the management of NSSI in individuals with ASD are discussed, including aspects such as persistence, severity, intervention strategies, and the functionality of NSSI behaviors. This structured presentation aims to facilitate a clear and systematic understanding of the findings.

### 3.1. Literature Search and Study Characteristics

**Figure 1:**
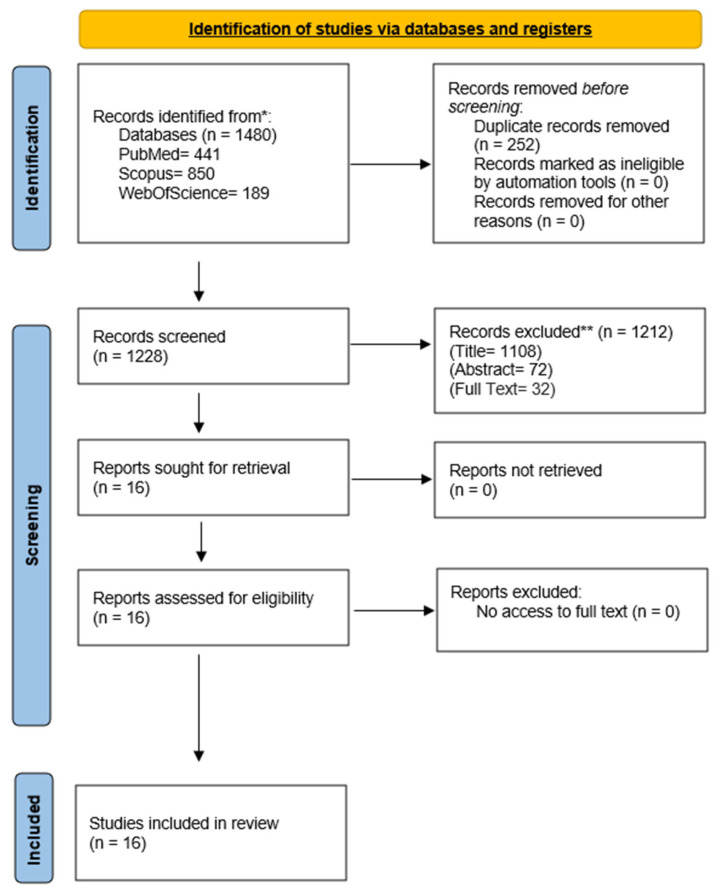
Flow diagram of non-suicidal self-injury in ASD. * Databases searched for records. ** Reasons for exclusion at full-text screening.

From among a total of 1480 articles identified by database searches, 1228 were finally selected for screening after removing duplicates. A general PRISMA flow chart was devised detailing the number of studies excluded at each stage of screening (Figure 1). Finally, 48 full-text articles were analyzed to assess their eligibility for the current systematic review. Only 16 articles met the inclusion criteria; therefore, they were included in the data extraction (Table 1 and Table 2) and quality assessment processes (Table 3, Table 4 and Table 5).

The selected studies were published between 2011 and 2024. Among the 16 studies, 14 were cross-sectional [17,18,21,22,23,24,25,26,27,28,29,30,31,32] and 2 prospective cohort studies [33,34]. Of the total selected studies, eight were conducted in Europe, most of them in the United Kingdom (UK) [18,25,29,33,34], but also in Spain [26], Ireland [27], and France [28]. Seven were conducted in the United States of America (USA) [17,22,23,24,30,31,32] and one in Pakistan [21].

The 16 articles that studied the factors associated with NSSI in ASD included 20,080 individuals. Of those, 16,425 were male. Two of the articles included a sample of adults (over 18) [17,18], seven studies only had minors in the sample [21,27,28,30,31,32,34], and seven included both age groups [22,23,24,25,26,29,33]. Only one article included an additional control group of participants with NSSI but without ASD [17]. The remaining studies compared participants diagnosed with ASD with and without NSSI.

### 3.2. Factors Associated with NSSI

This section is organized into six thematic domains: emotional, behavioral, cognitive, social, medical, and demographic factors. Each domain is analyzed in detail, highlighting specific variables and their relationship with the presence, frequency, and severity of NSSI in individuals with ASD. The findings are presented with a focus on identifying significant predictors and risk markers, as well as discussing inconsistencies across studies to provide a nuanced understanding of the multifaceted nature of NSSI in this population.

#### 3.2.1. Emotional Factors

Regarding emotional factors, significantly lower levels of anxiety and depression were associated with the absence of NSSI, compared to ASD groups that did exhibit these behaviors [18,30]. However, Maddox et al. [17] found no significant differences in depression levels between the NSSI and non-NSSI groups. Likewise, there appears to be a relationship between low mood and the presence of NSSI. Individuals with NSSI presented a significantly lower mood and this variable proved to be a significant predictor of the presence of NSSI [25,34]. Irritability was also found to be a statistically significant predictor of the frequency and severity of NSSI [22]. In addition, higher scores for irritability were found in the NSSI groups [24,28]. On the other hand, alexithymia was also found to be a significant predictor for the presence of NSSI. This difficulty in identifying and describing our own emotions was significantly higher in patients who were currently self-harming compared to those who were not [18]. No significant relationship was found between a history of NSSI and current levels of emotional dysregulation. However, in the subset of adults with ASD with NSSI, greater emotional dysregulation was associated with the presence of NSSI as a form of sensation seeking [17].

#### 3.2.2. Behavioral Factors

Behavior inhibition impairments were also associated with the presence of NSSI in individuals with ASD. Hyperactivity and impulsivity were widely associated with the presence of NSSI, showing significantly higher rates in groups that included participants diagnosed with ASD and NSSI [24,25,28,29,30,34]. Thus, both high levels of impulsivity and hyperactivity predicted a higher probability of severe NSSI in both children and adults [25,29]. Furthermore, impulsivity was found to be a predictor of NSSI persistence after a 3-year follow-up [34], and both variables, impulsivity and overactivity, were established as risk markers for NSSI persistence after a 10-year follow-up [33]. However, Gulsrud et al. [23] found no supporting evidence for hyperactivity as a factor associated with NSSI in ASD.

In addition, aggressive behaviors were also significantly associated with NSSI in individuals with ASD [22,30,31,32]. Regarding stereotypical and repetitive behaviors, evidence has also been found of their relationship with NSSI in patients with ASD [22,24,28,29,33,34], becoming a significant and independent predictor of the presence of NSSI only in the child sample and of the presence of severe NSSI in children and adults [29]. However, other studies do not support this relationship between NSSI and stereotyped or repetitive behavior [23,25].

#### 3.2.3. Cognitive Factors

Intelligence quotient (IQ) shows some controversy in its relationship with NSSI in autism. Some authors find a significant relationship between lower IQ scores and the presence of NSSI in both frequency and severity [22,24,27,28,32], with IQ being the only statistically significant predictor for NSSI [22,27]. In Gulsrud et al. [23], the association between IQ and NSSI had a medium to large effect size, although the relationship was not statistically significant. In contrast, Soke et al. [30] found no significant relationship between IQ and NSSI, and Soke et al. [31] even found a significant positive correlation between IQ and the presence of current NSSI, suggesting that greater intellectual capacity is associated with more self-harming behaviors. Other authors use adaptive capacity or ability as a way of measuring cognitive functioning. In this way, it was found that lower adaptive capacity and lower ability were associated with higher self-harm in individuals with ASD [21,28,29,34].

Moreover, sensory processing also does not yield consistent results. Some authors found that NSSI was related to sensory processing problems and sensory issues [30,31,32]. Specifically, Moseley et al. [18] found that sensory sensitivity was a predictor of the presence of NSSI. Specifically, sensory avoidance was associated with the number of areas of the body harmed and the incidence of NSSI throughout life, while sensory low registration was the only significant predictor of the frequency of NSSI. However, other authors did not find this association [22].

#### 3.2.4. Social Factors

Regarding social factors, NSSI was associated with deficits in communication skills in ASD [22,28,34], with these deficits being more severe depending on the persistence of NSSI in different contexts [24]. Regarding communication development, participants who engaged in NSSI showed a trajectory of low growth in communication skills [28]. Likewise, communication skills were identified as a protective factor against NSSI [28].

Furthermore, the presence of NSSI was also related to difficulties in social functioning [23,28] and difficulties in social interaction were important risk markers that can predict the persistence of NSSI in individuals with ASD over a 3-year period [34]. Despite the foregoing, Moseley et al. [18] found that deficits in mentalization were not associated with the presence or frequency of NSSI.

#### 3.2.5. Health Factors

In relation to the medical conditions of participants with ASD, some authors find an association between gastrointestinal problems and the presence of NSSI in child samples [29,31]. In contrast, Soke et al. [30] did not find this association. Richards et al. [29] demonstrated an association between NSSI and skin problems and found that childhood genetic conditions (i.e., Down syndrome, fragile X syndrome, tuberous sclerosis) were associated with current NSSI, although Rattaz et al. [28] do not confirm this finding. Sleep problems in child samples were also associated with the presence of NSSI [30,31,32]. Thus, medical problems could significantly increase the likelihood of NSSI in the child sample [29]. Additional mental health diagnoses were also reported in ASD groups with current or past NSSI compared to those without [18].

Prenatal, perinatal, and neonatal factors were examined in a small subset of the included studies [23,31,32]. In the SEED sample, perinatal and birth record-derived characteristics were associated with the presence of self-injurious behaviors in children with ASD, including maternal and delivery-related variables such as maternal age, cesarean delivery, and gestational age [31]. In addition, Soke et al. [32] identified several prenatal, perinatal, and neonatal complications, such as lower maternal educational attainment, prenatal maternal cigarette smoking, and the use of electronic fetal monitoring during labor, as significant risk factors for NSSI. Similarly, factors related to gestational age and birth weight were also relevant, with lower birth weight and prematurity or lower gestational age being associated with NSSI [23,32]. These perinatal markers are complemented by delays in early development, as individuals with NSSI were found to have delayed crawling age and delayed bladder and bowel control [23].

#### 3.2.6. Demographic Factors

The relationship between age and the prevalence or severity of NSSI in ASD is largely inconsistent. Most studies, which included both children and adults, have found no significant differences in the presence, severity, or type of self-harm [17,18,23,24,25,27,29,31]. Nevertheless, other studies provide evidence of possible cohort-specific risk patterns. The results of Soke et al. [30] significantly associated SIB with younger child age, while Akram et al. [21] found greater involvement in NSSI in adolescence (particularly in the 11–15 age group), indicating a possible window of vulnerability. This was also highlighted in Licence et al. [25], where, although the correlation was not significant, 60% of participants with NSSI were in the 12–18 age group. Interestingly, although age alone often lacks a strong linear correlation, in hierarchical regression analyses (adjusted for other variables), Flowers et al. [22] found that age can emerge as a significant predictor of the frequency and severity of SIBs, suggesting that its effect is complex and intrinsically linked to interaction with other risk factors.

The research also showed an inconsistent association between biological sex and the overall prevalence of NSSI in ASD. Most studies reported no significant differences in presentation or severity between males and females [22,24,25,27,28,29,30,34]. Despite this apparent agreement, other authors found significant gender differences. Akram et al. [21] and Maddox et al. [17] found that women showed more NSSI, while Soke et al. [31] found that male gender was associated with greater NSSI. On the other hand, Massaguer-Bardaji et al. [26] reported interesting results regarding the phenomenology and function of NSSI based on gender. Specifically, their findings indicated that women with ASD tended to self-harm more frequently through burning, writing letters on themselves, and pulling their hair. In addition, women were more likely to self-harm when alone and to do so to distance themselves from others, establish friendships, and express negative emotions in an uncontrollable manner.

Although the included studies reported mixed findings regarding sex differences in NSSI prevalence and severity, these results should be interpreted in the context of contemporary epidemiological and clinical evidence suggesting that autistic females may be at elevated risk for self-harm and suicidality. Population-based register data indicate that self-harm is strongly associated with subsequent suicide risk in autistic individuals of all sexes, while also suggesting that the relative impact of severe self-harm on suicide risk may be particularly pronounced among autistic females [35]. More broadly, recent syntheses highlight that suicidality risk in autism is substantial across the lifespan and may be especially elevated in autistic females without intellectual disability and in those with co-occurring conditions [36,37]. Importantly, apparent inconsistencies across ASD-focused NSSI studies may reflect methodological and ascertainment factors—such as under-recognition of autism in females, later diagnosis, and sampling bias—rather than the absence of meaningful sex-related differences. In this regard, diagnostic and social-contextual mechanisms (including camouflaging and unmet support needs) have been linked to increased suicidality risk in autistic adults, offering a plausible pathway through which cumulative distress may amplify vulnerability to NSSI and related outcomes in autistic females [38]. Taken together, these considerations support a more cautious interpretation of “inconsistent” sex effects and underscore the need for adequately powered, sex-stratified longitudinal studies using standardized definitions and measurement approaches.

Furthermore, ethnicity was a variable that was rarely studied in the studies. It did not appear to show a significant relationship with NSSI [24,30], although Soke et al. [31] found that children whose parents reported that they had ever engaged in NSSI were less likely to have a mother who belonged to a racial minority.

Similarly, in relation to parents and family context, NSSI was associated with lower maternal educational level [30,32], unmarried marital status [32], and younger mothers [31]. Regarding family socioeconomic status, no significant association was found with the presence of NSSI [28].

### 3.3. Clinical Implications and Management Challenges

NSSI in individuals with ASD represents a clinically significant and complex concern. The studies included in this systematic review reported NSSI prevalence rates ranging from 24% to 50%, indicating substantial variability across the literature. This heterogeneity may be attributable to differences in operational definitions, sample characteristics, and assessment methods. Establishing these prevalence estimates is essential for understanding the magnitude of the problem and for contextualizing findings related to NSSI persistence and remission in this population. These prevalence estimates provide the necessary context to interpret the available longitudinal evidence on persistence and remission.

Building on these baseline rates, the included studies suggest that NSSI tends to be persistent in a substantial proportion of cases, although remission may occur over longer timeframes. For example, Richards et al. [34] reported that NSSI persisted in 77.8% of participants with ASD over a three-year follow-up period, with no significant differences observed in the presence or topography of NSSI over time, suggesting relative stability in this interval. In contrast, Laverty et al. [33] found a 56% remission rate over a ten-year follow-up, with persistence in 44% of cases; notably, the only form of NSSI that showed significant reductions over time was self-biting. Together, these findings indicate that NSSI may follow a chronic course for many individuals, while remission remains possible in the long term and may vary across specific NSSI topographies.

The evidence also suggests that NSSI does not remit spontaneously, underscoring the importance of early intervention. Participants who received early-life intervention services showed a lower prevalence of NSSI compared with those who did not have access to such services [21]. In addition, parental involvement in behavioral management plans emerged as a protective factor and a negative predictor of NSSI [21], suggesting that active caregiver engagement may be crucial for reducing both the frequency and severity of self-injury. Findings related to treatment context should be interpreted cautiously: individuals currently receiving applied behavior analysis (ABA) reported higher frequency and severity of self-injury compared to those receiving eclectic approaches; however, this association likely reflects greater baseline clinical complexity in those receiving ABA rather than indicating that ABA constitutes a risk factor [27].

Regarding factors associated with NSSI persistence and remission, studies identified markers such as greater ASD symptom severity, the presence of restricted and repetitive behaviors (RRBs), impulsivity, and hyperactivity as significant risk indicators [25,33,34]. At the same time, evidence linking overall ASD severity to NSSI was mixed. While some studies reported no significant association between overall ASD severity and NSSI or found no differences in severity between self-harm groups [23,24,30], others identified greater symptomatic severity in individuals engaging in NSSI [21,28,33,34]. Moreover, specific symptom dimensions—such as insistence on sameness and difficulties in social interaction—were associated with NSSI both cross-sectionally and over time [25,33,34]. Conversely, adaptive functioning and communication abilities were identified as protective factors that may reduce the likelihood of persistent NSSI [28].

Beyond longitudinal course and associated factors, the available literature highlights substantial heterogeneity in the functional profile and clinical expression of NSSI in ASD. Several studies suggest that affective imbalance/emotion dysregulation is among the most prevalent reasons precipitating NSSI, followed by functions such as self-punishment, sensation seeking, and social communication or expression [17,18]. Similarly, Massaguer-Bardaji et al. [26] identified emotional self-regulation as the most frequently endorsed function, followed by the manifestation of distress, sensation seeking, and suicide avoidance. In terms of topography, common forms of NSSI include scratching or pinching oneself, hitting or banging oneself, self-beating, and cutting, although discrepancies between studies exist regarding their relative frequency [17,18,21,23,24,25,29]. The most frequently affected body areas included the arms, hands, head, and wrists [17,18]. Some studies reported that individuals engaged in multiple forms of NSSI [24,25], whereas others found that most participants exhibited a single form [23]. Severity appeared comparable to individuals without ASD [17] and remained stable over a three-year follow-up period [34]. High frequency patterns have also been reported, with 23.8% of individuals engaging in NSSI on more than 50 occasions [17] and 55.6% reporting at least weekly NSSI in one study [25]. Qualitative evidence further suggests that facilitating “understanding myself”—including emotion identification and expression, awareness of triggers, and learning coping strategies—may be particularly meaningful for treatment and self-management in autistic individuals [18].

In conclusion, NSSI in individuals with ASD is associated with concerning prevalence rates and appears to persist in a substantial proportion of cases, although remission may occur over longer periods and may differ by topography. The available evidence indicates that early intervention and parental support can play a critical role in reducing the frequency and severity of NSSI, while management should be guided by careful functional assessment and recognition of heterogeneity in both form and function. These findings highlight the importance of a comprehensive, individualized, and sustained clinical approach to address the specific needs of this vulnerable population.

**Table 1:** Main characteristics and findings of the analyzed studies.

Non-suicidal self-injury in ASD							
First Author (Publication Year), Study Name, Country	Objective	Study Design	Sample Size (N), Age (Mean ± age SD)	ASD Diagnostic Criteria	Instrument	Measured Variables	Results
McTiernan et al., 2011 [27]. Analysis of risk factors and early predictors of challenging behavior for children with autism spectrum disorder. Ireland.	To assess risk factors for the onset, frequency, and severity of problematic behaviors (such as self-injurious behavior, stereotyped behavior, and aggression).	Cross-sectional.	*N* = 174 children with ASD. (8 ± 2.38). 144 males and 30 females.	DSM-4-TR.	BPI-01 and Demographic questionnaire.	Age, Age at diagnosis, IQ, gender, type of intervention currently and at the time of intervention.	Lower IQ was a significant predictor of greater frequency and severity across all measured challenging behaviors. Current ABA interventions were associated with more severe stereotyped behaviors. Finally, participants who began with “eclectic” interventions at diagnosis scored significantly higher on the frequency and severity of self-injurious than those who started with ABA.
Rattaz et al., 2015 [28]. Symptom severity as a risk factor for self-injurious behaviours in adolescents with autism spectrum disorders. France.	To describe the prevalence of SIB and the relationship between SIB and clinical or environmental factors. The second objective was to identify risk factors for SIB among adolescents with ASD.	Cross-sectional.	*N* = 152 adolescent with ASD. 82.2% males. (15 ± 1.6).	World Health Organization research criteria.	ADI-R; ABC; PAR-DD-Qo; CARS; VABS; Wechsler intelligence scales, Early Social Communication Scale, test of pragmatic skills, demographic questionnaire, and medical reports.	Gender, SIB, ASD severity, irritability, lethargy, hyperactivity, stereotypy, adaptative behavior, object cognition, person cognition, total intervention, parent’s quality of life, verbal expressive language, parent’s socio-economic status, developmental trajectory in communication, drug use, and epilepsy.	35.8% of the total sample showed SIB. SIB was associated with irritability, stereotypy, and hyperactivity, severity of ASD, adaptive skills, intellectual functioning, and language level. Additionally, parents of adolescents with a high SIB reported less quality of life. The main risk factor for SIB at adolescence was the severity of autism both during adolescence and retrospectively in childhood. Higher cognitive development during childhood reduced the risk of SIB at adolescence. Having better communicative abilities was the main protective factor during adolescence.
Richards et al., 2016 [34]. Persistence of self-injurious behaviour in autism spectrum disorder over 3 years: a prospective cohort study of risk markers. UK.	To investigate the persistence of SIB in a cohort of individuals with ASD over three years and identify associated behavioral and demographic risk markers.	Prospective cohort study.	*N* = 67 with ASD Median (IQR) 10.00 (7.00–14.00) at T2. 85.1% male.	Confirmed diagnosis of ASD from a relevant professional.	Demographic questionnaire, The Wessex Behavior Scale; MIPQ-S; TAQ; SCQ; RBQ; CBQ.	Gender, age, ability, mobility, speech, vision, hearing, SIB, mood, interest and pleasure, overactivity, impulsivity, impulsive speech, stereotyped behavior, compulsive behavior, insistence on sameness, restricted preferences, communication, social interaction, and repetitive behavior.	SIB persisted in 77.8% of individuals with ASD over a 3-year period. Behavioral correlates of being non-verbal, having lower ability, and exhibiting higher levels of overactivity, impulsivity, and repetitive behavior were associated with SIB. Impulsivity and deficits in social interaction at baseline were identified as significant risk markers associated with the persistence of SIB. The presence, topographies, and severity of SIB were also found to be stable.
Akram et al., 2017 [21]. Prevalence and predictors of non-suicidal self-injury among children with autism spectrum disorder. Pakistan.	To find the prevalence and common forms of NSSI among children with ASD, and to identify both risk and protective factors predicting NSSI.	Cross-sectional.	*N* = 83 with ASD. (11.77 ± 3.59). 68% male.	Score on CARS.	ISAS and CARS.	NSSI, gender, age, severity of ASD, parental involvement in counselling, early intervention, and forms of NSSI.	The overall point prevalence of NSSI was 33%. The most common forms of self-harm were banging/self-beating (47%), scratching (38%), pinching (35%), picking scabs (33%), self-biting (32%), pulling hair (30%), and rubbing skin (19%). Regression analysis identified age, gender, and severity level of ASD as significant risk factors of NSSI. Conversely, early intervention and parental involvement in counselling emerged as significant protective factors against NSSI in this population.
Maddox et al., 2017 [17]. Untended wounds: Non-suicidal self-injury in adults with autism spectrum disorder. USA.	To examine NSSI, methods, frequency, severity, functions, and initial motivations in adults with ASD. The secondary aims were to compare NSSI characteristics between adults with and without ASD, and to explore the association of NSSI history with current depression symptoms and emotion regulation difficulties.	Cross-sectional.	*N* = 84. 21 ASD with NSSI, 21 ASD without NSSI 42 without ASD but with NSSI. ASD with NSSI (25.29 ± 7.77) ASD without NSSI (29.19 ± 10.87) NSSI without ASD (19.90 ± 1.43) 33.6% male.	Written report of ASD diagnosis.	NSSI-AT; DERS and Severity measure for depression-adult.	Method, severity, recency, frequency, location, and function of NSSI, gender, age, ethnicity, age at diagnosis, depression, emotional regulation, level of education, employment status, and residence status.	50% of adults with ASD reported a history of NSSI. Women with ASD were significantly more likely to endorse NSSI (72.2%) than men (33.3%). NSSI characteristics were generally similar to adults without ASD, but history of NSSI was not related to current depression or emotion dysregulation. A key difference was that ASD adults were significantly more likely to use NSSI to avoid committing suicide.
Richards et al., 2017 [29]. Predictors of Self-Injurious Behavior and Self-Restraint in Autism Spectrum Disorder: Towards a Hypothesis of Impaired Behavioral Control. UK.	To examine the associations between demographic and behavioral characteristics and SIB in children and adults with ASD, and the relationship between SIB and self-restraint in this population.	Cross-sectional.	*N* = 424 with ASD. (24.10 ± 13.01). 78.5% male.	Confirmed diagnosis of ASD from a relevant professional.	Demographic questionnaire; SIB; SAD-SQ; Self-Restraint Checklist; CBQ; Wessex Behavior Scale; activity questionnaire and the behavior and emotional difficulties section of the Self-Help and Behavior Rating Scale.	Gender, age, medication, contact with health professional, SIB, self-restraint, ability, painful health conditions, repetitive behavior, overactivity, and impulsivity.	SIB was highly prevalent (45.7% children; 49.1% adults) and significantly associated with self-restraint. The most frequent topography was hitting self with a body part. Severe SIB in children was predicted by lower ability, health problems, and overactivity/impulsivity. In adults, severe SIB was predicted by repetitive/restricted and overactive/impulsive behaviors. SIB and behavioral control indicators predicted self-restraint in children, while only SIB predicted it in adults.
Soke et al., 2017 [30]. Factors Associated with Self-Injurious Behaviors in Children with Autism Spectrum Disorder: Findings from Two Large National Samples. USA.	To assess factors associated with SIB considering child, parent, and family variables. To determine if any associations found were modified by child sex, IQ, or maternal education.	Cross-sectional.	*N* = 13,167 divided into two large samples with ASD. One subsample with 8-year-old participants and another with mean age 5.71 SD ± 3.4. 82.24% and 83.01% males, respectively.	DSM-4-TR.	Demographic survey; Stanford Binet Intelligence Scales-5th Edition Abbreviated Battery; MSEL; VABS.	Age, gender, ethnicity, IQ, adaptive skills, developmental regression, sleep and, sensory abnormalities, aggression, hyperactivity, attention problems, anxiety, mood problems, severity of ASD, parental age, maternal education, type of health insurance, and GI disturbances.	SIBs were associated with impaired adaptive behavior, developmental regression, aggression, hyperactivity, anxiety, mood problems, sensory abnormalities, and sleep problems. SIBs were also associated with younger child age and lower maternal education. SIBs were related to lower median census tract income and neurological conditions. No significant associations were found with child sex or race/ethnicity in either dataset, nor with gastrointestinal problems, ASD severity, or parental age.
Gulsrud et al., 2018 [23]. Self-injurious behaviours in children and adults with autism spectrum disorder (ASD). USA.	To utilize a relatively large, clinical sample of individuals with ASD across a wide range of age-independent variables to provide careful characterization of markers associated with the presence of SIB.	Cross-sectional.	*N* = 144 with ASD (9.3 ± 8.6). 81% males.	DSM-5	Demographics and medical history; ADI-R; ADOS-2; Wechsler intelligence scales; MSEL; Differential Ability Scales-II; SB-5; SRS-2; CBCL; BRIEF and BRIEF-P.	Age, gender, ethnicity, family background, school history, medical history, IQ, ASD symptoms, ability, speech, SIB (presence, severity, frequency), social impairment, emotional, executive functions, behavioral and social functioning, adaptive functioning, perinatal variables, and developmental milestones.	29.4% of the sample currently engaged in SIB, with head banging being the most common topography. Though not statistically significant after correction, the SIB group showed medium to large effect sizes for greater impairment in current cognitive (IQ) and social functioning. Early developmental delays, such as delayed crawling and later toileting, and perinatal risks like lower birth weight were also preliminarily associated with SIB.
Handen et al., 2018 [24]. Risk factors for self-injurious behavior in an inpatient psychiatric sample of children with autism spectrum disorder: A naturalistic observation study. USA.	To examine the rate and identify risk factors for SIB. Moreover, the study aimed to compare those who exhibited SIB across environments (home and hospital) with those who had SIB only at home, and to develop and validate clinically practical predictive models to identify which inpatient youth with ASD are most likely to engage in SIB.	Cross-sectional.	*N* = 302 with ASD. (12.9 ± 3.4) 78.81 males.	ADOS-2 cut-offs for ASD.	Leiter-3; VABS-2; ABC-C; RBSR; ADOS-2; SCQ; demographic questionnaire and observational recordings.	Age, gender, ethnicity, income, nonverbal IQ, ASD severity, verbal ability, irritability, lethargy, stereotypy, hyperactivity, social communication, inappropriate speech, and repetitive behavior.	67% of the sample presented home or hospital SIB. Neither ASD severity nor age or gender was found to be associated with SIB. The group with SIB across both home and hospital presented more severe and pervasive SIB and had significantly lower nonverbal IQ scores and higher scores on irritability, hyperactivity, and stereotypy subscales. Tree-structured modeling successfully generated two practical predictive models: one was highly accurate at predicting which youth with SIB at home would not continue SIB in the hospital, while the second was highly accurate at predicting which youth would have any SIB.
Soke et al., 2018 [31]. Self-injurious behaviors in children with autism spectrum disorder enrolled in the study to explore early development. USA.	To enhance knowledge of factors influencing SIB by assessing potential associations with both currently SIB and ever SIB, as reported in the ADI-R, in children with ASD. Additionally, the study aimed to evaluate the concordance between parental report of SIB and the clinician’s observations of SIB during the ADOS.	Cross-sectional.	*N* = 692 (55.97 ± 6.71) Age indicated in months. 81.79% males. Percentage calculated by researchers.	DSM-IV-TR.	ADOS; ADI-R; MSEL; VABS-2; CSHQ; GSI; caregiver interview; maternal medical history and birth certificates.	Current, ever and observed SIB, sociodemographic characteristics, developmental regression, IQ, adaptive score, autism severity, somatic conditions, child comorbid diagnoses, maternal medical and psychiatric conditions during pregnancy, and child perinatal conditions.	There was a discrepancy between the SIB observed in the ADOS and that reported in the ADI-R. Five variables (lower child adaptive skills, child sleep, gastrointestinal and behavioral problems/issues, and younger maternal age) were significantly associated with both current and ever SIB in multivariable models. Significant independent associations were also found between current SIB alone and higher cognitive skills, child genetic conditions, sensory problems, cesarean birth, and major neonatal complications. Variables significantly associated only with ever SIB included lower gestational age, male sex, and non-Hispanic white race.
Moseley et al., 2019 [18]. A ‘choice’, an ‘addiction’, a way ‘out of the lost’: exploring self-injury in autistic people without intellectual disability. UK.	To validate a previous descriptive analysis of NSSI in a larger autistic population without intellectual disability and to qualitatively analyze participants’ experiences with NSSI. Finally, the study intends to identify predictive factors for NSSI that may hold clinical relevance.	Cross-sectional.	*N* = 103 with ASD (43 ± 13.6). 32.04 males.	A formal diagnosis of autism.	NSSI-AT; TAS-20; AQ; RMET; BDI and BAI.	Age, age at diagnosis, comorbidity, alexithymia, ASD traits, sensory processing, mentalization, anxiety, depression, range, NSSI location, incidence, frequency, and function.	Alexithymia, depression, anxiety, and sensory sensitivity were significant predictors of being a current or historic self-harmer versus a non-self-harmer. The most common function of NSSI was regulating low-energy affective states (30%), followed by high-energy states (27%). Alexithymia also predicted NSSI use for high-energy state regulation and communication, while sensory differences predicted NSSI range, incidence, and frequency.
Soke et al., 2019 [32]. Prenatal, perinatal, and neonatal factors associated with self-injurious behaviors in children with autism spectrum disorder. USA.	To explore potential associations between SIB and prenatal, perinatal, and neonatal factors identified from birth certificates, and validate associations between SIB and developmental, behavioral, and medical factors.	Cross-sectional.	*N* = 4343 with ASD 8 years old. 82.5% males.	DSM-IV-TR.	Record-based surveillance system.	Gender, gestational age, birth weight, Apgar score, maternal and paternal age, mother’s education and marital status, maternal smoking, maternal weight gain during pregnancy, delivery, type of pregnancy, labor complications, obstetric procedures, developmental regression, IQ, sleep and sensory problems.	Significant associations between SIB and three perinatal factors: lower maternal educational attainment, prenatal maternal cigarette smoking, and the use of electronic fetal monitoring during labor. Furthermore, the study validated previous associations between SIB and various developmental, behavioral, and medical factors, confirming that children with SIB were significantly more likely to exhibit developmental regression, lower IQ, aggression, argumentative behaviors, temper tantrums, and sensory and sleep problems.
Licence et al., 2020 [25]. Prevalence and risk-markers of self-harm in autistic children and adults. UK.	To describe self-harm prevalence, forms, and severity in autistic children and adults without adaptive impairments. It also investigated associations with demographic factors, autism severity, age of diagnosis, and behavioral risk markers like impulsivity, repetitive behaviors, and affect.	Cross-sectional.	*N* = 83 with ASD. (14.13 ± 6.2) 80.7% male.	Confirmed diagnosis of ASD from a relevant professional.	Demographic questionnaire; Wessex Behavior Scale; mood; MIPQ-S; TAQ; RBQ; SCQ; CBQ.	Presence and topography of self-harm, demographic characteristics, autism severity, age of diagnosis, affect, impulsivity, overactivity, and repetitive behavior.	The study found a relatively high self-harm prevalence of 24.1% in autistic individuals without adaptive impairments. Common forms included hitting self (60%), biting self (50%), and scratching (50%). Self-harm was significantly associated with higher impulsivity, overactivity, compulsive behavior, insistence on sameness, and lower mood. Low mood and overactivity/impulsivity were identified as significant predictors, correctly classifying 82.9% of cases.
Flowers et al., 2020 [22]. Associated factors of self-injury among adolescents with autism spectrum disorder in a community and residential treatment setting. USA.	To analyze client records of children with ASD to describe the distribution of SIB and health disorders; to examine the relationships between SIB, other repetitive behaviors, and adaptive skills; and to identify demographic, psychological, behavioral, and health factors that predict SIB.	Cross-sectional.	*N* = 145 with ASD (16 ± 3.26) 79% male.	DSM-5.	BPI-01; ABC; ASRS and Vineland-II Composite Standard Score.	SIB frequency and severity, age, gender, aggression, stereotypies, irritability, cognitive functioning, adaptive skills (socialization, life skills, and communication), and medical variables.	50% of all participants displayed both high SIB frequency and severity. Regression analysis consistently identified irritability and cognitive functioning as significant predictors for both SIB frequency and severity. Specifically, better cognitive functioning resulted in a 2% decrease in SIB frequency and severity. Age was a significant predictor in some models, but, medical conditions were not found to predict SIB.
Laverty et al., 2020 [33]. Persistence and predictors of self-injurious behaviour in autism: a ten-year prospective cohort study. UK.	To investigate SIB in individuals with ASD over a 10-year period, specifically to study the persistence of SIB and identify behavioral and demographic characteristics associated with persistent SIB. Furthermore, the study sought to develop a predictive model for SIB.	Prospective cohort study.	*N* = 67 with ASD (23.9 ± 7.7) 80.6% male.	Confirmed diagnosis of ASD from a relevant professional.	Demographic questionnaire; Wessex Behavior Scale; TAQ; SCQ; RBQ; CBQ and the Self-Restraint Questionnaire.	Age, gender, ability, mobility, speech, autism phenomenology, type of SIB, impulsivity, hyperactivity, adaptive functioning, repetitive behavior, and demographic characteristics of parents.	Self-injury was persistent in 44% of individuals over the 10-year period with a significant reduction in overall SIB from T1 to T3. Behavioral characteristics of impulsivity and overactivity were identified as robust risk markers, strongly predicting both the cross-sectional presence and the 10-year persistence of self-injury and self-restraint. A predictive model for self-injury persistence included baseline impulsivity, interest and pleasure, stereotyped behavior, social communication, and adaptive functioning.
Massaguer-Bardaji et al., 2024 [26]. Differences in self-harm among adolescents and young adults with autism spectrum disorder: a gender-based approach. Spain.	To explore whether there is significant gender differences among adolescents diagnosed with autism who have been admitted to the neurodevelopment unit at ITA Argentona due to NSSI.	Cross-sectional.	*N* = 50 with ASD Age range between 14 and 27 years. The mean age and SD were not specified. 56% male.	Previous confirmed diagnosis, ADOS-2 and ADI-R.	ADI-R; ADOS-2 and ISAS.	Gender, age, IQ, suicide attempts, types of NSSI, motivations and functions for NSSI, incidence of NSSI, and tendency to be alone when self-harming.	The study found no substantially greater likelihood of one sex engaging in NSSI. However, significant gender differences were identified in specific types of self-harm and associated motivations. Autistic women were more prone to NSSI involving burning, carving letters, and hair pulling. Furthermore, women showed a greater tendency to self-harm to regulate strong emotions, create friend bonds, distance themselves from others, and to hurt loved ones.

**Abbreviations:** ABC (Aberrant Behavior Checklist), ABC-C (Aberrant Behavior Checklist-Community), ADI-R (Autism Diagnostic Interview-Revised), ADOS (Autism Diagnostic Observation Schedule), ADOS-2 (Autism Diagnostic Observation Schedule, Second Edition), AQ (Autism-Spectrum Quotient), ASD (Autism Spectrum Disorder), ASRS (Sensory subscale on the Autism Spectrum Rating System), BAI (Beck Anxiety Inventory), BDI (Beck Depression Inventory), BPI-01 (Behavior Problems Inventory), BRIEF (Behavior Rating Inventory of Executive Function), BRIEF-P (Behavior Rating Inventory of Executive Function-Preschool Version), CARS (Childhood Autism Rating Scale), CBCL (Child Behavior Checklist), CBQ (Challenging Behavior Questionnaire), CSHQ (Child Sleep Habit Questionnaire), DERS (Difficulties in Emotion Regulation Scale), GSI (Gastrointestinal Symptom Inventory), ISAS (Inventory of Statements About Self-Injury), Leiter-3 (Leiter International Performance Scale-Third Edition), MIPQ-S (Mood Interest and Pleasure Questionnaire-Short Form), MSEL (Mullen Scales of Early Learning), NSSI (Non-Suicidal Self-Injury), NSSI-AT (Non-Suicidal Self-Injury Assessment Tool), RBQ (Repetitive Behavior Questionnaire), RBSR (Repetitive Behavior Scale-Revised), RMET (Adolescent-Adult Sensory Profile, “Reading the Mind in the Eyes” Test), SAD-SQ (Aggression and Destruction Screening Questionnaire), SB-5 (Stanford–Binet Intelligence Scales: Fifth Edition), SCQ (Social Communication Questionnaire), SRS-2 (Social Responsiveness Scale, Second Edition), TAQ (Activity Questionnaire), TAS-20 (Toronto Alexithymia Scale), VABS (Vineland Adaptive Behavior Scale), and VABS-2 (Vineland Adaptive Behavior Scales-2).

**Table 2:** Correlates of NSSI/SIB in ASD across six domains.

Study	Emotional	Behavioral	Cognitive	Social	Medical	Demographic/Perinatal
McTiernan et al., 2011 [27].	—	More severe challenging behaviors in those currently receiving ABA; higher SIB frequency/severity in those who started with “eclectic” interventions.	Lower IQ; greater frequency/severity of challenging behaviors.	—	—	No association with age or gender.
Rattaz et al., 2015 [28].	Irritability (associated).	Hyperactivity; stereotypy (associated).	Lower adaptive skills; lower intellectual functioning (associated).	Lower language/ communication level. More risk in low-growth trajectories.	No differences in medical condition. Increased drug use among adolescents (risk factor). ASD severity (risk factor).	No association with gender or socioeconomic status.
Richards et al., 2016 [34].	Lower mood (risk factor).	Overactivity, impulsivity; stereotyped and compulsive behavior (risk markers). Stability in the topography of NSSI over time.	Lower ability (associated).	Non-verbal status and deficits in social interaction (risk factor).	ASD severity (risk factor).	No association with gender.
Akram et al., 2017 [21].	—	—	—	Parental involvement and early intervention (protective factors).	ASD severity (risk factor).	Age and gender (risk factors).
Maddox et al., 2017 [17].	NSSI history not related to current depression.	The most damaged parts of the body were the arms and hands. Affective imbalance as the most common reason for NSSI.	—	—	—	No association with age. Gender (risk factor).
Richards et al., 2017 [29].	—	Overactivity/impulsivity and Repetitive/restricted behaviors (predictors of severe SIB in children).	Lower ability (predictor of severe SIB in children).	—	Health problems (predictors in children).	No association with age or gender.
Soke et al., 2017 [30].	Anxiety (associated).	Aggression; hyperactivity (associated).	No association with IQ. Sensory abnormalities (associated).	—	No associations with gastrointestinal problems or genetic conditions. Sleep problems (associated).	No association with gender or ethnicity. Younger child age; lower maternal education (associated).
Gulsrud et al., 2018 [23].	—	No association with RRBs or hyperactivity.	Greater cognitive impairment/IQ differences. early developmental delays (crawling, toileting).	Greater social impairment (effect sizes).	No association with ASD severity.	No association with age. Perinatal risks (lower birth weight; preliminary).
Handen et al., 2018 [24].	Higher irritability (associated)	Hyperactivity and stereotypy behavior (associated)	Lower non-verbal IQ (more severe/pervasive SIB group).	More social and communication deficits in SIB group	No association with ASD severity.	No association with age, gender or ethnicity
Soke et al., 2018 [31].	—	Behavioral problems/issues (associated).	Lower adaptive skills (associated); higher cognitive skills (associated with current SIB only). Sensory problems (associated)	—	Sleep problems, gastrointestinal problems, genetic conditions (associated).	No association with age. Male sex and ethnicity associated. Perinatal risks (Younger maternal age, cesarean birth, neonatal complications and lower gestational age)
Moseley et al., 2019 [18].	Alexithymia (predictor), depression, anxiety (associated).	The most damaged parts of the body were the arms and hands. Affective imbalance as the most common reason for NSSI.	Sensory sensitivity (associated).	Mentalization not associated.		No association with age.
Soke et al., 2019 [32].	—	Aggression and temper tantrums (associated).	Developmental regression, lower IQ and sensory problems (associated).	—	Sleep problems (associated).	Lower maternal education; prenatal smoking; electronic fetal monitoring during labor (associated).
Licence et al., 2020 [25].	Lower mood (associated and predictor).	Overactivity/impulsivity, compulsive behavior and insistence on sameness (associated/predictors). Most SIBs were reported weekly or more frequently.	—	—	—	No association with age or gender.
Flowers et al., 2020 [22].	Irritability (predictor).	Aggression and stereotypies (associated).	IQ (predictor).	Communication skills (associated).	Medical conditions not predictive.	Age significant in some models. No association with gender.
Laverty et al., 2020 [33].	Interest and pleasure (included in persistence model).	Stereotyped behavior. Impulsivity, overactivity (predictors of persistence). Significant reductions in presence and topography over time.	Adaptive functioning (included in persistence model).	Social communication (included in persistence model).	More access to pediatricians in SIB group.	—
Massaguer-Bardaji et al., 2024 [26].		—	—	—	—	No overall sex difference in likelihood of NSSI; gender differences in motivations/function.

**Notes:** Correlates are reported as described in the included studies and grouped into six domains (emotional, behavioral, cognitive, social, medical, and demographic/perinatal). “—” indicates that the domain was not assessed or no relevant findings were reported for that study. **Abbreviations**: ABA (Applied Behavior Analysis), ASD (Autism Spectrum Disorder), IQ (Intelligence Quotient), NSSI (Non-Suicidal Self-Injury), RRBs (Restricted and Repetitive Behaviors), SIB (Self-Injurious Behavior).

### 3.4. Risk of Bias Assessment and Study Limitations

The methodological quality of the included studies was predominantly favorable: 12 studies were classified as having a low risk of bias [18,21,22,23,24,25,27,28,29,30,31,32], three studies as having a moderate risk of bias [17,33,34], and one study as having a high risk of bias [26]. For the study-level breakdown by design and appraisal tool, see Table 3, Table 4 and Table 5.

Related to the studies’ limitations, while recent studies have increasingly elucidated the characteristics and predictors of NSSI in the general population, there remains a significant gap in research specifically addressing this phenomenon in individuals with ASD. Traditionally, the ASD population has been explicitly excluded from NSSI studies. This exclusion is rooted in earlier conceptualizations that considered SIB in ASD as a single clinical entity, and only as a form of RRB. In consequence, the role of NSSI as an emotional regulation strategy may have been frequently overlooked by both researchers and clinicians, as autistic social communication impairments may limit the explanation of function or purpose of self-harm. Furthermore, the interpretation of the results is hindered by the lack of standardized terminology, as SIB, RRB, and NSSI are often used interchangeably or defined inconsistently. On the other hand, available research on NSSI in ASD considers gender differences from a dichotomous model, without considering gender incongruence in individuals with ASD [39,40]. Future studies could consider gender incongruence or gender dysphoria as possible variables that influence the presence of NSSI.

Another important limitation is the heterogeneity of the studies included, concerning the diagnostic criteria for ASD, with consistent changes between DSM-4 and DSM-5 [41,42]. In addition, the autism spectrum itself includes a wide variety of clinical symptoms, making it difficult to compare individuals with this diagnosis. Finally, there is also considerable heterogeneity in the methods used to assess the variables included, which may also hinder comparisons between studies.

**Table 3:** Risk of bias assessment of studies analyzed.

Study	Design	Risk of Bias Tool	Overall Risk of Bias
McTiernan et al., 2011 [27].	Cross-sectional	JBI Checklist-Cross-sectional	Low risk
Rattaz et al., 2015 [28].	Cross-sectional	JBI Checklist-Cross-sectional	Low risk
Richards et al., 2016 [34].	Cohort study	JBI Checklist-Cohort Studies	Moderate risk
Akram et al., 2017 [21].	Cross-sectional	JBI Checklist-Cross-sectional	Low risk
Maddox et al., 2017 [17].	Cross-sectional	JBI Checklist-Cross-sectional	Moderate risk
Richards et al., 2017 [29].	Cross-sectional	JBI Checklist-Cross-sectional	Low risk
Soke et al., 2017 [30].	Cross-sectional	JBI Checklist-Cross-sectional	Low risk
Gulsrud et al., 2018 [23].	Cross-sectional	JBI Checklist-Cross-sectional	Low risk
Handen et al., 2018 [24].	Cross-sectional	JBI Checklist-Cross-sectional	Low risk
Soke et al., 2018 [31].	Cross-sectional	JBI Checklist-Cross-sectional	Low risk
Moseley et al., 2019 [18].	Cross-sectional	JBI Checklist-Cross-sectional	Low risk
Soke et al., 2019 [32].	Cross-sectional	JBI Checklist-Cross-sectional	Low risk
Licence et al., 2020 [25].	Cross-sectional	JBI Checklist-Cross-sectional	Low risk
Flowers et al., 2020 [22].	Cross-sectional	JBI Checklist-Cross-sectional	Low risk
Laverty et al., 2020 [33].	Cohort study	JBI Checklist-Cohort Studies	Moderate risk
Massaguer-Bardaji et al., 2024 [26].	Cross-sectional	JBI Checklist-Cross-sectional	High risk

**Note:** Study quality/risk of bias was assessed using the Joanna Briggs Institute (JBI) Critical Appraisal Checklist for Analytical Cross-Sectional Studies (8 items), covering key domains such as sample selection, measurement validity/reliability, confounding, and appropriateness of statistical analysis. Items were rated Yes/No/Unclear/NA; only “Yes” was counted as met. Overall quality was classified as high risk/low quality (0–3), moderate risk/moderate quality (4–6), or low risk/high quality (7–8) based on the number of criteria met.

**Table 4:** JBI checklist item ratings for included cross-sectional studies.

Study	1. Clearly Defined Inclusion Criteria.	2. Detailed Description of Subjects and Setting.	3. Exposure Measured in a Valid and Reliable Way.	4. Objective and Standard Criteria for Measuring the Condition.	5. Identified Confounding Factors.	6. Strategies for Addressing Confounding Factors.	7. Results Measured in a Valid and Reliable Way.	8. Appropriate Statistical Analysis.
McTiernan et al., 2011 [27].	Yes	Yes	Yes	Yes	Yes	Yes	Yes	Yes
Rattaz et al., 2015 [28].	Yes	Yes	Yes	Yes	Yes	Yes	Yes	Yes
Akram et al., 2017 [21].	Yes	Yes	Yes	Yes	Yes	Yes	Yes	Yes
Maddox et al., 2017 [17].	Yes	Yes	Yes	Yes	Yes	Yes	No	Yes
Richards et al., 2017 [29].	Yes	Yes	Yes	Yes	Yes	Yes	Yes	Yes
Soke et al., 2017 [30].	Yes	Yes	Yes	Yes	Yes	Yes	Yes	Yes
Gulsrud et al., 2018 [23].	Yes	Yes	Yes	Yes	Yes	Unclear	Yes	Yes
Handen et al., 2018 [24].	Yes	Yes	Yes	Yes	Yes	Unclear	Yes	Unclear
Soke et al., 2018 [31].	Yes	Yes	Yes	Yes	Yes	Yes	Yes	Yes
Moseley et al., 2019 [18].	Yes	Yes	Yes	Yes	Yes	Yes	Yes	Yes
Soke et al., 2019 [32].	Yes	Yes	Yes	Yes	Yes	Yes	Yes	Yes
Licence et al., 2020 [25].	Yes	Yes	Unclear	Yes	Yes	Yes	Yes	Yes
Flowers et al., 2020 [22].	Yes	Yes	Yes	Yes	Yes	Yes	Yes	Yes
Massaguer-Bardaji et al., 2024 [26].	Yes	Yes	Yes	Yes	Unclear	Unclear	No	Unclear

**Note:** Study quality/risk of bias was assessed using the Joanna Briggs Institute (JBI) Critical Appraisal Checklist for Analytical Cross-Sectional Studies (8 items), covering key domains such as sample selection, measurement validity/reliability, confounding, and appropriateness of statistical analysis. Items were rated Yes/No/Unclear/NA; only “Yes” was counted as met. Overall quality was classified as high risk/low quality (0–4), moderate risk/moderate quality (5–7), or low risk/high quality (8) based on the number of criteria met.

**Table 5:** JBI checklist item ratings for included cohort study studies.

Study	1. Similar Groups Recruited from the Same Population.	2. Exposure Measured in a Similar Way to Assign People to Groups.	3. Exposure Measured in a Valid and Reliable Way.	4. Identified Confounding Factors.	5. Strategies for Addressing Confounding Factors.	6. Participants Free from the Outcome at the Start.	7. Outcomes Measured in a Valid and Reliable Way.	8. Follow-Up Time Reported.	9. Follow- Up Completed.	10. Strategies for Addressing Incomplete Follow-Up.	11. Appropriate Statistical Analysis.
Richards et al., 2016 [34].	Yes	Yes	Yes	Yes	Yes	No	Yes	Yes	Yes	Unclear	Yes
Laverty et al., 2020 [33].	Yes	Yes	Yes	Yes	Yes	No	Yes	Yes	Yes	Unclear	Yes

**Note:** Study quality/risk of bias was assessed using the Joanna Briggs Institute (JBI) Critical Appraisal Checklist for Analytical Cohort study Studies (11 items), covering key domains such as group formation, exposure measurement, confounding factors, and statistical analysis used. Items were rated Yes/No/Unclear/NA; only “Yes” was counted as met. Overall quality was classified as high risk/low quality (0–6), moderate risk/moderate quality (7–9), or low risk/high quality (10–11) based on the number of criteria met.

## 4. Discussion

This systematic review synthesizes the current evidence on NSSI in individuals with ASD and identifies a set of multidomain factors consistently associated with NSSI, alongside key challenges for clinical management and correlates of NSSI in ASD within emerging neurobiological models. Across studies, the findings underscore that NSSI in ASD is best understood as a complex, multifactorial phenomenon shaped by emotional, behavioral, cognitive, social, medical, and demographic correlates. Importantly, the available evidence suggests that NSSI may serve heterogeneous functions and can present with varying severity, chronicity, and topography, highlighting the need for individualized clinical formulation and sustained monitoring in high-risk presentations. Taken together, these findings provide a framework to interpret NSSI in ASD beyond a single explanatory mechanism and to guide clinically actionable assessment targets.

A central contribution of this review is the clarification of a persistent conceptual gap in the ASD self-injury literature. Prior research has frequently focused on SIB within the framework of RRBs, while NSSI has often been under-recognized as a distinct construct. The frequent interchangeability of terms such as “SIB”, “self-harm”, and “NSSI” has likely contributed to conceptual ambiguity, limiting comparability across studies and constraining the translation of findings into clinical practice. By explicitly distinguishing between NSSI, SIB, and RRBs, this review provides a more precise framework for interpreting self-injury phenotypes in ASD and for aligning assessment approaches with the functional characteristics of the behavior.

Based on this conceptual distinction, the review offers the first structured synthesis of correlates of NSSI in ASD across six domains—emotional, behavioral, cognitive, social, medical, and demographic—thereby organizing a fragmented evidence base into a clinically meaningful framework. In parallel, the review highlights practical complexities that may complicate intervention delivery, including persistence over time, variability in severity and physical presentation, and functional heterogeneity (e.g., emotion regulation, sensory modulation, self-punishment, and communicative functions). Together, these findings support the clinical utility of function-based assessment and underscore the relevance of tailoring intervention strategies to individual profiles, rather than assuming a uniform mechanism of self-injury across autistic individuals. In the following sections, we discuss these domains in relation to prior literature and highlight their implications for clinical formulation and intervention planning.

At the same time, the synthesis reveals important limitations in the existing literature, including methodological heterogeneity, inconsistencies in terminology and operationalization, and uneven coverage of specific risk factors. In particular, the limited consideration of gender incongruence and prenatal/perinatal influences represents a notable gap that warrants more systematic investigation. The following discussion situates the present findings within prior research, examines their implications for clinical assessment and intervention planning, and outlines priorities for future work to strengthen the evidence base and improve clinical management of NSSI in ASD.

The heterogeneity in clinical presentation of ASD, coupled with its high rates of comorbidities that may increase internal psychopathological burden, lead to specific risk behaviors in these patients, such as SIB, which includes NSSI. In this line, it is crucial to distinguish SIB—often characterized by rhythmic and repetitive actions, typically linked to severe intellectual disability or considered a form of RRB—from NSSI. Nevertheless, both NSSI and RRB have been described as a mechanism of stress regulation [9,17].

SIB, in the context of ASD, is typically prevalent among those individuals with severe or profound intellectual disability, lower levels of adaptive behavioral functioning, and significant deficits in receptive and/or expressive communication. Nevertheless, these specific characteristics are not recognized as primary risk factors for NSSI in non-autistic population [43]. This contrast in associated risk profiles highlights the need to differentiate between SIB and NSSI within ASD.

Several studies found an association between RRB (as a form of SIB) and NSSI in ASD patients [22,24,28,29,33,34]. Specifically, in one of these studies [29], RRB were found to be a significant and independent predictor of the presence of NSSI in the child sample, and of the presence of severe NSSI in both children and adults. Conversely, other studies did not support a significant relationship between NSSI and RRB [23,25]. This inconsistency across findings may be attributed to differences and potential inaccuracies in how SIB, RRB, and NSSI are conceptualized and operationalized across studies.

The collected evidence suggests that NSSI in ASD is frequently associated with affective imbalance and emotion-regulation needs [17,18,26], consistent with dominant functional models of NSSI described in the broader literature [44,45] and in non-clinical populations as well [3,46]. In non-autistic samples, converging findings from self-report and phenomenological studies indicate that negative affect commonly precedes self-injury and is followed by perceived relief, supporting affect regulation as one of the most robustly documented mechanisms of NSSI [44]. In line with this, the reviewed ASD-focused studies also describe emotion regulation and affective imbalance as common precipitants of NSSI [17,18,26], suggesting partial convergence in both underlying motivations and behavioral methods across populations. Indeed, there is similarity in the methods of self-harm reported by individuals with ASD and those without this diagnosis, including scratching or pinching, hitting or banging oneself, self-beating, and cutting [17,18,21,23,24,25,29,44,45,46,47,48], and this pattern is consistent with findings in non-autistic samples in which most participants report self-injury as a strategy to alleviate negative emotions [44,45,46,47,48].

At the same time, the strength of the conclusion that NSSI in ASD is “driven” by affective imbalance warrants cautious interpretation. The current evidence base is characterized by methodological heterogeneity and is predominantly cross-sectional, with variability in the operationalization of NSSI and in the measurement of emotional constructs. Accordingly, affective imbalance is best understood as one of the most consistently reported correlates and functions of NSSI in ASD [17,18,26], rather than a single primary causal driver. This interpretation supports a more integrative conceptualization in which NSSI in ASD is likely multifactorial, reflecting interactions between emotional, cognitive, social-communication, sensory, and medical factors that may influence onset, maintenance, and clinical presentation [18].

Within the broader context of affective dysregulation, alexithymia—defined as difficulty in identifying and describing one’s own emotions and present in approximately half of individuals with ASD [9]—emerges as a particularly relevant vulnerability factor. In our included studies, alexithymia was significantly higher among individuals currently engaging in self-harm compared to those who were not [25], suggesting that NSSI may function as a compensatory strategy when verbal labeling, differentiation, or communication of distress is impaired. Meta-analytic evidence further indicates that alexithymia is highly prevalent in autistic individuals and may characterize a substantial subgroup within ASD [47], reinforcing its potential clinical relevance for understanding NSSI vulnerability. Importantly, qualitative accounts also suggest that sensory differences and heightened physiological arousal may co-occur with affective distress and contribute to NSSI risk, raising the possibility that NSSI may serve not only affect-regulation purposes but also sensory modulation or relief from overwhelming internal states in some individuals [18].

From a clinical perspective, these findings support individualized, function-based assessment that explicitly evaluates emotion identification and expression (including alexithymia), alongside potential sensory triggers and co-occurring psychiatric or medical factors that may amplify affective instability. Taken together, the evidence is most consistent with a model in which affective imbalance represents a central—but not exclusive—pathway to NSSI in ASD [17,18,26], and highlights the need for longitudinal studies and methodologically standardized approaches to better delineate the mechanisms and interactions underpinning NSSI in this population. Clinically, this supports prioritizing function-based formulations that integrate emotional, sensory, social-communication, and medical contributors when developing individualized management plans.

In addition, the relationship between NSSI and sensory processing abnormalities, a core characteristic of ASD [6], introduces a specific etiological link in this population. Sensory alterations are a source of significant distress for individuals with ASD. These manifestations typically present in two distinct patterns: on the one hand, some individuals with ASD may experience low auditory, visual, tactile, or olfactory thresholds. In these cases, common stimuli, such as bright lights or background noise, become overwhelming and cause discomfort. Conversely, some may experience a decrease in sensory registration. This diminished sensitivity to internal or external stimuli—such as pain, heat, or hunger—can be life-threatening, as the individual may not recognize or respond to physical injury or physiological needs [7]. As results showed, NSSI was related to sensory processing problems and sensory issues [30,31,32]. Specifically, Moseley et al. [18]. found that sensory sensitivity was a predictor of the presence of NSSI. For its part, sensory avoidance was associated with the number of areas of the body harmed and the incidence of NSSI throughout life, while sensory low registration was the only significant predictor of the frequency of NSSI. However, other authors did not find this association [22].

In addition, sensory processing avoidance may be related to the absence of pain perception or the lack of recognition of other underlying health problems. Evidence suggests that comorbid medical problems, such as gastrointestinal issues [29,30,31], skin problems [29], and sleep problems [30,31,32], were associated with NSSI presence. These medical issues, potentially masked by atypical sensory thresholds or by the difficulty of individuals with ASD to communicate internal discomfort, could precipitate NSSI as a dysfunctional way of manifesting bodily pain or physiological distress. For instance, clinicians should avoid automatically attributing self-injurious or aggressive behaviors displayed by autistic individuals to a primary psychiatric or behavioral disorder without first thoroughly evaluating potential underlying medical causes. Greater awareness is essential as disruptive behaviors in individuals with ASD, including self-injury and aggression, may represent manifestations of physical pain or discomfort [49]. This finding has direct implications for assessment pathways, as it emphasizes the need for routine screening of potential medical contributors when NSSI is present.

In alignment with literature from non-autistic samples, which links NSSI to a higher psychopathology—like depression, anxiety [3,50,51], and other mental disorders, such as borderline personality disorder [52,53,54]—research in ASD population shows a comparable trend. On the one hand, results found internalizing distress, particularly low mood, as a robust predictor for the presence of self-harm [25,34]. On the other hand, externalizing distress (specifically irritability) was found to be a significant predictor of both the frequency and severity of these behaviors [22,24,28].

In addition, it has been reported that hyperactivity and impulsivity were widely linked to NSSI as well [24,25,28,29,30,34]. Moreover, aggressive behaviors were consistently and significantly associated with NSSI engagement in individuals with ASD across multiple studies [22,30,31,32]. This correlation is not casual, as the prevalence of ADHD in ASD populations is substantial, with a recent study showing rates of 28% for ADHD and 11% for disruptive, impulse-control, and conduct disorders [55].

Given that the core symptomatology of ASD includes impairment in social interactions [6], it is essential to analyze the association between these deficits and NSSI. Not surprisingly, results found that NSSI is linked to difficulties in communication skills in individuals with ASD [22,28,34], and better communication skills were identified as a protective factor against NSSI [28]. Furthermore, the presence of NSSI was also related to difficulties in social functioning [23,28], and difficulties in social interaction were identified as important risk markers to predict the persistence of NSSI in individuals with ASD [34]. Such results are consistent with broader literature on non-clinical and non-autistic samples that describe NSSI related to alterations in processing of social-emotional stimuli [56,57]. Nonetheless, despite the foregoing evidence linking social impairment to NSSI, one study found that deficits in mentalization, among the most characteristic issues in the ASD population that affects social interactions and relationships, were not found to be associated with the presence or frequency of NSSI [18].

NSSI is a critical public health concern, with peak prevalence occurring during adolescence and young adulthood, across both clinical and non-clinical populations [3,17]. Although prevalence of NSSI in non-autistic population has been reported to be higher in adolescents (17.2%) and young adults (13.4%) [58], the relationship between age and the prevalence of NSSI in ASD remains inconsistent. Most studies, which included both children and adults, found no significant differences in the presence, severity, or type of self-harm [17,18,23,24,25,27,29,31]. Conversely, other studies suggest potential cohort-specific risk patterns. For instance, Soke et al. [30] significantly associated SIB with younger child age. In contrast, others indicated a higher involvement in NSSI during adolescence (the 11–18 age range), suggesting this might be a period of heightened risk [21,25]. Although age alone often lacks a strong linear correlation, hierarchical regression analyses found that age can emerge as a significant predictor of the frequency and severity of SIB when adjusted for interaction with other risk factors [22].

The severity of NSSI in individuals with ASD appears to be similar to that observed in individuals without this disorder [17] and tends to remain stable over time [34]. The synthesis of results on the clinical management of NSSI suggests that self-harm does not remit spontaneously, highlighting the importance of early intervention, as participants who received intervention at an early stage showed less NSSI compared to those who did not receive services in their early years [21]. Additionally, parental involvement in behavioral management plans emerges as a protective factor and negative predictor of NSSI [21]. These findings reinforce early access to services and caregiver involvement as pragmatic targets for prevention and longer-term risk reduction.

Based on the multifactorial framework described above, sex- and gender-related variables may shape not only the prevalence of NSSI but also its phenomenology, function, and clinical presentation. In this context, examining gender-linked patterns is clinically relevant, as it may reflect differentiated pathways to affective dysregulation, social stress, and coping strategies in ASD. The research showed that biological sex was not predominantly associated with the overall prevalence of NSSI in ASD, as most of them reported no significant differences in presentation or severity between males and females [22,24,25,27,28,29,30,34]. These results are consistent with prior research on non-clinical populations as well. However, prevalence estimates alone may fail to capture clinically meaningful differences in how NSSI manifests and is maintained across individuals. For instance, a systematic review that analyzed prevalence of NSSI in non-clinical samples, found that NSSI occurred at similar rates regardless of gender. This may be explained by the historical view of NSSI as a predominantly female issue, likely stemming from studies focused on psychiatric inpatients, with a concentration of female patients diagnosed with borderline personality disorder, a condition where self-harm is a primary diagnostic marker. In addition, gender differences in self-injury methods—specifically the female preference for cutting versus the male tendency toward self-battery—suggest that prior research that limited NSSI to cutting, underestimated the incidence of NSSI in male populations [58].

Nevertheless, Massaguer-Bardaji et al. [26] highlighted substantial differences in the phenomenology and function of NSSI in individuals with ASD based on gender: women with ASD tended to use self-harm more frequently through burning, writing letters on themselves, and pulling their hair, and were more likely to self-harm when alone, to distance themselves from others, to use self-harm to establish friendships, and to express negative emotions in an uncontrollable manner. These findings could be related to the camouflaging phenomena, which is described as the ability to observe and imitate peers and to mimic their behaviors. It involves masking autistic characteristics to better fit into social situations, often leading not only in delayed detection or misdiagnosis of ASD, but also in a significant psychological burden on the individual, contributing to increased levels of internalizing distress [10,12,59]. This phenomenon is particularly present in individuals with high cognitive ability. As higher cognitive abilities are more frequent in women with ASD, camouflaging is also more prevalent in that gender [12].

The emotional exhaustion secondary to camouflaging is further compounded by the fact that autistic women experience higher rates of psychiatric comorbidities—including anxiety, depression, and eating disorders—which are frequently misidentified, and so, improperly treated [12]. Consequently, the sustained effort of social masking, alongside the mismanagement of co-occurring conditions, can lead to acute emotional dysregulation (such as outbreaks of anger) or social withdrawal when overstressed [59]. Within this context of chronic distress, NSSI may emerge as a maladaptive coping strategy to ameliorate overwhelming internal tension. In this context, it is also important to consider that social-communication difficulties may not only reflect individual impairment but may arise from cumulative contextual stressors, particularly in individuals who engage in sustained social camouflaging (masking) and are vulnerable to burnout-related exhaustion.

In fact, beyond being conceptualized solely as individual-level impairments, social-communication difficulties in ASD can also be understood as contextual stressors shaped by cumulative environmental demands, social invalidation, and sensory overload. Contemporary autism research increasingly emphasizes the role of autistic masking (camouflaging)—that is, sustained compensatory strategies aimed at concealing autistic traits to navigate social contexts—which has been described as particularly salient among autistic girls and women [10,12,59]. Prolonged masking has been associated with heightened internalizing distress, emotional exhaustion, and reduced access to supportive responses [60,61]. In the same line, the construct of autistic burnout—characterized by pervasive exhaustion, loss of functioning, and increased sensitivity to stressors following prolonged demands—has gained recognition as a clinically meaningful crisis state that may amplify affective vulnerability [62]. From this perspective, persistent social stress and invalidation may intensify emotional dysregulation and overwhelm coping resources, thereby increasing the likelihood that NSSI is enacted as a last-resort regulatory strategy to reduce acute internal tension, manage sensory-affective overload, or regain a sense of control. This contextual framing provides a plausible explanatory bridge between the social-communication correlates identified in the reviewed studies and the observed heterogeneity in NSSI persistence and severity, particularly in individuals experiencing chronic exhaustion or burnout-related deterioration. Notably, while these mechanisms extend beyond the systematically reviewed evidence, they offer a theoretically informed framework that may guide future longitudinal research examining how masking, burnout, and cumulative environmental stress interact with emotion regulation and sensory processing to shape NSSI trajectories in ASD.

Finally, the association between prenatal and perinatal complications and ASD has been extensively studied [63]. Large population studies have shown that low birth weight, complications during delivery, and neonatal complications are significantly associated with a diagnosis of ASD [64]. Within the scope of the present review, prenatal and perinatal factors were also reported in association with NSSI among individuals with ASD in a limited number of included studies [23,31,32]. However, the current evidence remains insufficient to determine the directionality, specificity, or mechanisms underlying these associations. Therefore, rather than indicating a confirmed etiological pathway, these findings should be interpreted as preliminary and hypothesis-generating, highlighting the need for future longitudinal research to clarify whether early perinatal adversities contribute to more vulnerable clinical profiles within ASD that may increase the likelihood of later difficulties in emotional and behavioral regulation and, consequently, NSSI engagement.

While this systematic review provides valuable insights into the factors associated with NSSI in individuals with ASD, several limitations should be acknowledged. First, we acknowledge that our search strategy, which included the exclusion term (“NOT suicid*”), may have inadvertently omitted studies reporting both suicidal and non-suicidal self-injurious behaviors, potentially limiting the scope of the evidence captured. However, this decision was intentionally made to align with the primary objective of the review, namely to focus specifically on NSSI and its associated correlates in ASD, and to minimize conceptual overlap with suicidal phenomena. To reduce this risk while preserving the specificity of the review, we applied inclusion criteria requiring that self-harming behaviors were explicitly described as occurring without suicidal intent or that studies provided sufficient operationalization to distinguish NSSI from suicidal behaviors. In addition to these search-related constraints, the predominance of cross-sectional designs limits inferences regarding temporal ordering and causality across identified correlates.

Additionally, it is important to note that methodological variability across studies may have influenced the strength of the conclusions that can be drawn. The heterogeneity of the included studies, particularly in terms of diagnostic criteria for ASD and the operationalization of NSSI, SIB, and RRB, poses challenges for direct comparisons and synthesis of results. The lack of standardized terminology and methodologies across studies further complicates the interpretation of findings. Moreover, the exclusion of studies addressing gender incongruence and the limited exploration of prenatal and perinatal factors highlight gaps in the current literature that warrant further investigation. Future research should aim to address these limitations by adopting more inclusive search strategies, standardized definitions, and methodologies to enhance the comparability and generalizability of findings.

Notwithstanding these limitations, this review is timely because it addresses a substantial gap in the literature on NSSI among individuals with ASD. To date, research on self-injury in ASD has largely focused on SIB conceptualized as part of RRBs, frequently overlooking NSSI as a distinct clinical phenomenon. Consequently, NSSI has remained underexplored as a potential strategy for emotion regulation in autistic individuals. To address this gap, the review systematically synthesizes evidence on factors associated with NSSI in individuals with a formal ASD diagnosis, organizing findings across six domains: emotional, behavioral, cognitive, social, medical, and demographic. It also discusses key challenges for clinical management, including the chronicity, severity, and functional characteristics of NSSI, thereby informing more targeted prevention and intervention strategies. Overall, this synthesis strengthens the clinical interpretability of the evidence by mapping correlates and management challenges within a unified multidomain framework.

Furthermore, the review underscores the need for standardized terminology to clearly differentiate SIB, RRBs, and NSSI, as these constructs are often used interchangeably in research and clinical contexts, potentially leading to conceptual and interpretive inconsistencies. Greater conceptual precision would improve comparability across studies and strengthen the clinical translation of findings by supporting more accurate assessment, case formulation, and intervention planning.

Finally, the review identifies critical avenues for future research—particularly the roles of gender incongruence and prenatal/perinatal factors—which remain largely neglected in the study of NSSI in ASD. In addition, future research should employ longitudinal designs and standardized measures to clarify the temporal relationship between affective imbalance and NSSI in ASD, and to examine how emotional dysregulation interacts with sensory processing differences, social-communication difficulties, and medical comorbidities to influence NSSI onset and maintenance. Such advances would enable more precise identification of modifiable mechanisms and support the development of targeted, evidence-informed interventions.

By clarifying these conceptual and empirical gaps, this review contributes to a more nuanced understanding of NSSI in ASD and supports the development of improved clinical management and tailored interventions for this vulnerable population.

### 4.1. Correlates of NSSI in ASD Within Emerging Neurobiological Models

To extend the conceptual scope of the present synthesis, it is also relevant to situate the correlates identified across studies within emerging neurobiological frameworks that conceptualize NSSI in ASD as a downstream manifestation of altered stress physiology and atypical interoceptive–autonomic processing. Notably, the neurobiological literature was not systematically reviewed in the present work; therefore, the following section should be interpreted as an integrative contextual framework that may help to generate mechanistic hypotheses and guide future empirical research, rather than as evidence derived from the included studies. Within this interpretive context, converging models propose that emotional overload and escalating arousal states in ASD may be shaped by autonomic dysregulation—characterized by heightened sympathetic activation and/or reduced parasympathetic regulation—thereby increasing vulnerability to impulsive and repetitive self-injury during distress [65]. Recent psychophysiological research also highlights the growing potential of objective autonomic markers (e.g., heart rate variability indices) to support identification of dysregulated arousal states in ASD, which may ultimately inform monitoring and prevention strategies for high-risk behavioral episodes [66,67].

From this perspective, the multidomain correlates synthesized in the present review—particularly affective imbalance, alexithymia, sensory sensitivity/avoidance, and medical comorbidity—may be understood as interacting components of a broader interoceptive–allostatic vulnerability profile. Interoception-based accounts of autism increasingly emphasize that atypical processing of internal bodily signals (e.g., pain, visceral cues, physiological arousal) may contribute to difficulties labeling emotional states and regulating distress, with the insula and related salience-network circuitry often highlighted as key neurobiological hubs [68]. This framework offers a plausible mechanism through which alexithymia and emotion-identification deficits—frequently reported in association with NSSI in autistic individuals—may reflect impaired mapping between bodily arousal and subjective emotional awareness, increasing reliance on behavioral strategies such as NSSI to modulate or externalize internal distress. Similarly, the consistent relevance of sensory processing differences in the reviewed studies can be conceptually aligned with models in which heightened arousal, atypical pain/interoceptive integration, and sensory overload contribute to self-injury not only as affect regulation but also as sensory modulation or relief from overwhelming physiological activation [68]. Taken together, these emerging neurobiological models complement the present findings by supporting a more integrative formulation in which NSSI in ASD reflects the interplay between emotional dysregulation, autonomic arousal dynamics, interoceptive processing differences, and contextual stressors, underscoring the need for longitudinal and methodologically standardized studies to test these mechanistic pathways directly.

From a clinical perspective, this neurobiological framing may help refine assessment and intervention priorities without reducing NSSI to a purely behavioral coping strategy. Specifically, heightened arousal and autonomic instability provide a mechanistic rationale for incorporating strategies that strengthen emotion regulation and distress tolerance, alongside individualized identification of sensory triggers and environmental stressors. In practice, this supports comprehensive, function-based case formulation that integrates emotional and sensory profiles, evaluates impulsivity and broader behavioral dysregulation, and systematically rules out or treats contributory medical conditions that may amplify physiological distress. Moreover, the prospective value of objective autonomic monitoring (e.g., heart rate variability or electrodermal indices) warrants further investigation as a complement to clinical judgement in detecting early signs of escalation and informing prevention planning in individuals at elevated risk [66,67]. The consistent relevance of sensory processing differences in NSSI further supports the clinical value of integrating neurobiologically informed formulations into assessment and intervention planning, as considering heightened arousal, sensory overload, and atypical pain/interoceptive integration may facilitate more tailored strategies that address both affect regulation and sensory modulation needs.

### 4.2. Clinical Implications for Assessment and Intervention

The findings of this systematic review support the need for targeted and individualized approaches to the clinical management of NSSI in individuals with ASD. Given the marked heterogeneity in presentation and function, clinical decision-making should be guided by function-based assessment and formulation, integrating emotional, behavioral, sensory, social-communication, and medical contributors to NSSI [17,18,26]. In particular, early identification appears clinically relevant, as access to intervention services during the early stages of development has been associated with lower prevalence of self-harm and may reduce the likelihood of persistence over time [21]. Accordingly, practitioners should consider proactive screening for NSSI and related risk markers in autistic individuals, especially during developmental periods of heightened vulnerability.

Caregiver involvement also emerges as a key element of clinical management. Parental participation in behavioral management plans has been identified as a protective factor and negative predictor of NSSI [21], suggesting that treatment approaches may benefit from actively engaging families through psychoeducation and skills-based guidance. Alongside caregiver support, behavioral intervention planning should be tailored to the functional characteristics of NSSI. Although some findings indicate that individuals receiving applied behavior analysis (ABA) present with higher frequency and severity of self-injury [27], this association should be interpreted with caution, as it likely reflects greater baseline clinical complexity among those referred to ABA rather than indicating that ABA constitutes a risk factor. Consequently, practitioners should ensure that behavioral plans explicitly address NSSI through individualized functional assessment, while coordinating multidisciplinary input when risk is severe, persistent, or associated with significant impairment.

A further consideration is the likely systematic under-detection of NSSI in autistic individuals, which may have influenced both prevalence estimates and the strength of observed associations. Emerging work indicates substantial discrepancies between self-report measures and clinician-administered interviews in autism, with some individuals endorsing self-harm and suicidal thoughts on questionnaires while denying these experiences during face-to-face assessment [69]. This measurement gap may reflect autism-related communication differences, difficulties identifying and verbalizing internal states (e.g., alexithymia), fear of negative consequences following disclosure, and limited clinician familiarity with autism-specific presentations of self-harm [70,71]. In parallel, concerns have been raised regarding the validity and acceptability of standard suicide/self-harm screening tools when applied to autistic populations, supporting the need for adapted instruments and multimethod assessment strategies [72,73].

From a clinical perspective, these limitations underscore the relevance of proactive and autism-informed detection pathways, including the combined use of self-report questionnaires and structured clinical interviewing, caregiver/teacher input when appropriate, and communication accommodations (e.g., visual supports, alternative response formats, additional time, and explicit normalization of disclosure). Strengthening assessment procedures may reduce false negatives and improve early identification of risk states, thereby supporting timely intervention and more accurate case management in autistic individuals presenting with suspected NSSI [70,73].

Moreover, from an affective perspective, the reviewed studies consistently position emotion-regulation needs as a prominent correlate and functional pathway of NSSI in ASD [17,18,26]. This highlights the importance of assessing emotional processes that may increase vulnerability, particularly alexithymia, which has been linked to current self-harm engagement [18] and may hinder the identification and communication of distress. Interventions that strengthen emotional awareness, labeling, and adaptive regulation skills may therefore be clinically valuable when NSSI serves intrapersonal regulatory functions. At the same time, sensory and medical contributors warrant systematic attention in clinical assessment. Sensory processing abnormalities, which are a core feature of ASD [6], have been associated with NSSI in several studies [18,30,31,32], suggesting that sensory sensitivity, avoidance, or low registration may contribute to distress and behavioral dysregulation in some individuals. In parallel, comorbid medical conditions—including gastrointestinal difficulties [29,30,31], skin problems [29], and sleep disturbances [30,31,32]—may increase discomfort and exacerbate risk, particularly when internal states are difficult to communicate. In this context, clinicians should avoid automatically attributing NSSI to psychiatric or behavioral causes without first evaluating potentially treatable medical contributors [49]. Finally, although biological sex is not consistently associated with overall NSSI prevalence [22,24,25,27,28,29,30,34], differences in phenomenology and function have been reported [26], reinforcing the need for individualized case formulation. Given that NSSI may persist over time and does not appear to remit spontaneously [34], sustained monitoring and follow-up are recommended, particularly for presentations characterized by greater severity, chronicity, or multi-method self-injury.

Beyond these findings, the present review underscores the need to strengthen clinical capacity through targeted professional training in the assessment and management of NSSI in ASD. Given the heterogeneity of mechanisms and presentations, mental health and neurodevelopmental practitioners should be equipped to conduct integrated, function-based case formulation that explicitly considers emotion regulation difficulties, sensory processing differences, social-communication stressors, and potentially contributory medical comorbidities. Such training is essential to improve early identification, refine risk stratification, and support the implementation of individualized intervention strategies that reduce preventable clinical burden.

Based on these management considerations, contemporary clinical practice increasingly emphasizes autism-adapted and neurodiversity-affirming intervention pathways that translate these findings into more actionable care. Although the approaches outlined below extend beyond the systematically reviewed evidence base, they provide clinically relevant context for applying the present results in routine practice.

From the perspective of clinical management, the present findings support a shift from descriptive risk profiling toward individualized, function-based, and autism-informed care pathways. Given that NSSI in ASD is frequently associated with affective dysregulation, impulsivity, sensory processing differences, and social-communication stressors, clinical assessment should move beyond symptom counting and instead prioritize formulation of the proximal function of self-injury (e.g., relief of overwhelming arousal, affect regulation, sensory modulation, self-punishment, or communicative signaling). This approach aligns with contemporary neurodevelopmental models emphasizing heterogeneity and the importance of matching intervention targets to the individual’s regulatory profile, contextual demands, and co-occurring conditions.

With respect to the practical translation of these findings into intervention strategies, contemporary practice increasingly highlights the clinical value of autism-adapted psychotherapies, particularly when emotion regulation difficulties and alexithymia are salient. Dialectical behavior therapy (DBT), adapted for autistic individuals, has shown feasibility and acceptability and may reduce emotion dysregulation in autistic adults presenting with self-harm and/or suicidal behaviors [74,75]. These adaptations typically emphasize concrete skills coaching, structured sessions, and accommodations for autism-related communication and sensory needs, thereby improving accessibility and engagement. In parallel, sensory-informed approaches may be particularly relevant in individuals whose NSSI appears linked to sensory overload, interoceptive distress, or atypical pain processing. Occupational therapy models incorporating sensory integration principles have been examined in ASD and have shown benefits for modulation and behavioral regulation in some cohorts, although evidence remains heterogeneous and intervention selection should be individualized [76,77]. In practice, this supports integrating proactive sensory profiling, environmental modification, and regulation strategies (e.g., graded sensory input, predictable routines, recovery planning following overload) alongside psychological interventions.

Notably, the review’s social-communication findings also have direct implications for clinical pathways, particularly where NSSI may function to express distress in the context of limited verbalization, fear of consequences, or reduced access to supportive responses. In such cases, incorporating augmentative and alternative communication (AAC) and functionally oriented communication supports may reduce reliance on self-injury as a maladaptive signaling strategy by strengthening alternative routes for expressing needs and requesting breaks or support [78,79]. Clinically, this underscores the value of multimodal assessment (self-report measures, structured clinical interviewing, and caregiver/teacher input when appropriate), combined with autism-sensitive disclosure practices and communication accommodations, to reduce false negatives and improve early identification of NSSI.

Finally, these clinical recommendations are increasingly situated within neurodiversity-affirming frameworks, which emphasize collaborative goal setting, respect for autistic experience, and the prioritization of well-being and adaptive functioning over normalization goals. Neurodiversity-affirming approaches encourage clinicians to interpret self-injury within the person’s lived context, strengths, and sensory–cognitive profile, and to design supports that reduce environmental mismatch and cumulative stress while enhancing autonomy and effective coping [80,81]. Current debates support an integrated model of clinical care in which NSSI in ASD is addressed through coordinated, multidisciplinary, and individualized strategies that combine function-based assessment, autism-adapted psychotherapy, sensory-informed planning, communication supports, and sustained monitoring for persistent or severe presentations.

From a broader perspective, reducing the prevalence and severity of NSSI in ASD should be a priority for prevention-oriented services and health policy. Early intervention programs and structured caregiver support represent promising, scalable targets for risk mitigation, particularly during sensitive developmental periods such as adolescence. Future research should advance the field by adopting longitudinal and methodologically standardized designs capable of clarifying temporal pathways and interactive mechanisms, while addressing underexplored domains including gender incongruence and prenatal/perinatal influences. Finally, increasing the visibility of NSSI within ASD is not only a clinical imperative but also an ethical and societal one, requiring greater awareness, empathy, and sustained support for autistic individuals for whom self-injury may reflect complex emotional and contextual distress.

## 5. Conclusions

To sum up, this systematic review underscores the multifaceted nature of NSSI in individuals with ASD and highlights the need for a more nuanced and clinically actionable conceptualization of this phenomenon. Overall, the findings indicate that NSSI in ASD is shaped by a complex interplay of emotional, behavioral, cognitive, social, medical, and demographic factors. Emotional dysregulation emerges as a central—but not exclusive—pathway to NSSI, with correlates such as alexithymia, depression, anxiety, and irritability consistently linked to NSSI onset and maintenance, suggesting that self-injury may frequently serve an affect-regulatory function. In parallel, behavioral profiles characterized by impulsivity, hyperactivity, and aggression appear strongly associated with NSSI, reinforcing the relevance of targeting behavioral dysregulation within individualized intervention planning. Cognitive functioning also contributes to vulnerability, as lower IQ and reduced adaptive capacity are related to higher prevalence and severity of self-injury in some studies. Notably, sensory processing abnormalities—including sensitivity and avoidance—represent significant predictors of NSSI, indicating that sensory modulation and distress related to sensory input may constitute clinically meaningful mechanisms for a subset of individuals. Social and communication impairments further contribute to risk, particularly as markers of persistence, emphasizing that NSSI may be embedded within broader challenges in emotional expression, social interaction, and help-seeking. Finally, associations with medical conditions—most notably gastrointestinal and sleep problems—suggest that physical discomfort and unmet physiological needs may act as precipitating or maintaining factors and should be systematically considered in clinical management. This pattern partially converges with functional models of NSSI described in broader non-autistic populations, supporting affect regulation as a common mechanism while emphasizing ASD-relevant interacting contributors. In addition, emerging neurobiological accounts provide a complementary interpretive context, suggesting that dysregulated autonomic arousal and atypical interoceptive processing may amplify vulnerability to escalating distress states in which NSSI functions as a rapid regulatory strategy in some individuals with ASD.

From the perspective of clinical practice, the evidence suggests that NSSI in ASD rarely remits spontaneously and may persist over time, underscoring the relevance of sustained monitoring and long-term management strategies, especially for severe or recurrent presentations. Early intervention and caregiver involvement emerge as meaningful protective factors, supporting proactive screening and timely access to services as key priorities to reduce both the likelihood of persistence and overall clinical burden. Although overall prevalence does not consistently differ by biological sex, differences in phenomenology and function highlight the need for individualized and gender-sensitive assessment, particularly given evidence that autistic women may engage in specific forms of self-harm and may use NSSI in relation to emotional regulation and social processes. Adolescence may also represent a period of heightened vulnerability, reinforcing the need for developmentally informed prevention and intervention approaches. Importantly, systematic under-detection and barriers to disclosure may further complicate both prevalence estimates and clinical management, reinforcing the value of autism-informed assessment pathways that combine multi-method screening, communication accommodations, and collateral informant input when appropriate.

In sum, these findings support the clinical utility of function-based assessment approaches that integrate emotional, sensory, social-communication, behavioral, and medical contributors when developing individualized formulations and intervention plans. This integrative perspective also supports contemporary intervention approaches, including autism-adapted psychotherapies targeting emotion regulation, sensory-informed planning to reduce overload and support regulation, and augmentative or alternative communication strategies when NSSI serves communicative or help-seeking functions. They also emphasize the need for greater conceptual precision in differentiating NSSI from SIB and restricted and repetitive behaviors (RRBs), as inconsistent terminology continues to constrain both research comparability and clinical translation. Future research should prioritize longitudinal designs and standardized methodologies to clarify temporal relationships and causal mechanisms, while also addressing underexplored domains such as gender incongruence and prenatal/perinatal influences. Further work should also evaluate the clinical utility of objective physiological and behavioral markers of escalating arousal (e.g., autonomic indices) to support earlier detection and prevention of high-risk episodes. Advancing this evidence base will be essential for improving risk stratification, refining assessment strategies, and developing more targeted and effective interventions for autistic individuals who engage in NSSI.

## Data Availability

The data that support the findings of this study are available on request from the corresponding author.

## Supplementary Materials

The following supporting information can be downloaded at: https://www.mdpi.com/article/10.3390/jcm15031254/s1, Table S1: PRISMA 2020 checklist.
